# Stepwise reprogramming of liver cells to a pancreas progenitor state by the transcriptional regulator Tgif2

**DOI:** 10.1038/ncomms14127

**Published:** 2017-02-13

**Authors:** Nuria Cerdá-Esteban, Heike Naumann, Silvia Ruzittu, Nancy Mah, Igor M. Pongrac, Corinna Cozzitorto, Angela Hommel, Miguel A. Andrade-Navarro, Ezio Bonifacio, Francesca M. Spagnoli

**Affiliations:** 1 Laboratory of Molecular and Cellular Basis of Embryonic Development, Max Delbrück Center for Molecular Medicine, Robert-Rössle-Str. 10, Berlin 13092, Germany; 2 Computational Biology and Data Mining, Max Delbrück Center for Molecular Medicine, Robert-Rössle-Strasse 10, Berlin 13092, Germany; 3 BCRT, Charité University Medicine Berlin, Augustenburger Platz 1, Berlin 13353, Germany; 4 DFG-Center for Regenerative Therapies Dresden, Technische Universität Dresden, Fetscher Straße 105, Dresden 01307, Germany; 5 Faculty of Biology, Johannes Gutenberg University Mainz and Institute of Molecular Biology, Ackermannweg 4, Mainz 55128, Germany

## Abstract

The development of a successful lineage reprogramming strategy of liver to pancreas holds promises for the treatment and potential cure of diabetes. The liver is an ideal tissue source for generating pancreatic cells, because of its close developmental origin with the pancreas and its regenerative ability. Yet, the molecular bases of hepatic and pancreatic cellular plasticity are still poorly understood. Here, we report that the TALE homeoprotein TGIF2 acts as a developmental regulator of the pancreas versus liver fate decision and is sufficient to elicit liver-to-pancreas fate conversion both *ex vivo* and *in vivo*. Hepatocytes expressing *Tgif2* undergo extensive transcriptional remodelling, which represses the original hepatic identity and, over time, induces a pancreatic progenitor-like phenotype. Consistently, *in vivo* forced expression of *Tgif2* activates pancreatic progenitor genes in adult mouse hepatocytes. This study uncovers the reprogramming activity of TGIF2 and suggests a stepwise reprogramming paradigm, whereby a ‘lineage-restricted' dedifferentiation step precedes the identity switch.

## Abstract (web summary)

Liver and pancreas cells arise from a common endoderm progenitor in the embryo, but what regulates their cell fate is unclear. Here, the authors show that expression of the Three-Amino-acid-Loop-Extension (TALE) homeobox TG-interacting factor 2 (TGIF2) in hepatocytes reprogrammes the cells to a pancreatic fate.

Successful lineage reprogramming relies on the identification of defined factor(s) able to establish the new cell fate transcriptional program and, concomitantly, silence the original gene expression program^1^,^2^,^3^,^4^. Here, we sought to investigate cellular plasticity between liver and pancreas and to what extent this enables their fate interconversion.

Lineage reprogramming holds distinct advantages over stem cell-based replacement strategies, with the new cells being autologous in origin, residing within their native tissue, and with a theoretically lower risk of tumorigenesis^5^. Recent studies have unveiled an unsuspected degree of cellular plasticity in the adult pancreas and pointed to pancreas-resident cells as potential sources for new β-cells^6^,^7^,^8^,^9^,^10^,^11^,^12^,^13^,^14^,^15^. However, from a clinical perspective, adult liver cells hold important advantages over pancreatic cells, representing a more accessible and abundant starting cell population for fate conversion approaches to generate pancreatic cells with therapeutic potential^3^,^16^. To date, adenovirus-mediated ectopic expression of pancreatic transcription factors (TF) (for example, *Pdx1*, *Ngn3*) in the liver has been shown to partially correct hyperglycaemia in diabetic mice, even though the phenotypic conversion appears incomplete^17^,^18^,^19^,^20^. Moreover, delivery of the same pancreatic TFs through adeno-associated viruses (AAV), which have low-immunogenity but high-transduction efficiency, or upon transgene expression in mice did not exert the same effects^19^,^21^,^22^. These observations are consistent with the fact that the two lineages are closely related but yet at a greater distance than cells of the same lineage, for example, exocrine and endocrine pancreas cells^23^,^24^. Additional limitations might be the lack of appropriate interaction partners or the presence of antagonistic factors in liver cells that lock cell identity, hampering cell plasticity and conversion. To overcome these lineage restrictions, we investigated whether developmental regulator(s) of the pancreas versus liver fate decision might be effective reprogramming determinants for achieving conversion of liver cells into pancreas fate.

The transcriptional mechanisms underlying hepatic and pancreatic lineage divergence in the mouse embryo begin to be unravelled^25^,^26^,^27^. In a RNASeq gene expression profile of liver and pancreas progenitor cells isolated from the mouse embryo, we identified the Three-Amino-acid-Loop-Extension (TALE) homeobox TG-interacting factor 2 (TGIF2) at the cell-fate branchpoint, being in the common endoderm progenitor pool and whose expression changes in opposite directions as cells commit to pancreatic or hepatic lineages^25^,^28^. TALE proteins are involved in many crucial developmental processes and diseases through their function as Hox-transcriptional co-regulators, but also through Hox-independent mechanisms^29^,^30^. TGIF2 contains a highly conserved homeodomain and has been described as a context-independent transcriptional repressor, exerting its function either through modulation of the BMP/TGF-beta pathway or through direct DNA binding^28^,^31^. In *Xenopus* embryos, Tgif2 acts as an intracellular endodermal effector promoting pancreatic fate by inhibiting BMP signalling^28^. In the mouse embryo, overlapping functions between *Tgif2* and its close family member, *Tgif1*, have been previously reported during different embryological processes, such as gastrulation and neural development^32^,^33^.

Here, we demonstrate that TGIF2 is a developmental regulator of pancreas versus liver fate decision using both loss- and gain-of function experiments in mouse embryos and embryonic stem (ES) cells, respectively. In addition, when ectopically expressed in murine adult liver cells, TGIF2 promotes suppression of the hepatic transcriptional program and induction of a subset of pancreatic genes. Overall, hepatocytes expressing *Tgif2* acquire a pancreatic progenitor state and upon exposure to pancreatic microenvironment or *in vivo* transplantation into diabetic mice the reprogrammed cells undergo further differentiation and acquire certain functional pancreatic properties. Similarly, *in vivo* AAV-mediated *Tgif2* expression in adult mice turns on marker genes of the pancreatic lineage in hepatocytes. In summary, this study defines a novel strategy for controlled generation of pancreatic progenitors based on TGIF2-dependent fate conversion and opens to new investigation into the mechanistic aspects of cellular identity and plasticity.

## Results

### Liver and pancreas fate divergence

**Figure 1:**
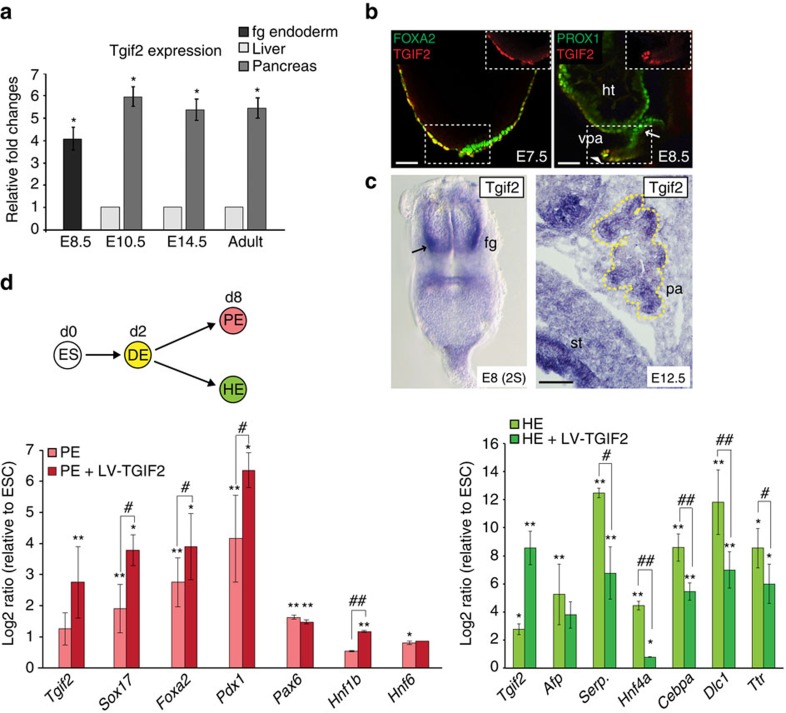
TGIF2 controls pancreatic and hepatic cell lineage divergence. (**a**) RT-qPCR analysis of *Tgif2* expression in the mouse foregut (fg) endoderm and its derivatives, liver and pancreas. Data were normalized to that of *Sdha* and represented as fold change (FC) compared with liver samples (set to 1). E8.5 fg was compared with E10.5 liver sample. Values shown are mean±s.e.m. (*n*=3) *P<0.05. (**b**) TGIF2 (red) colocalizes with FOXA2 (green) in the E7.5 mouse anterior DE and with PROX1 (green) in the E8.5 at the fg lip (see arrowhead). Arrow indicates prospective hepatic endoderm (HE). Inset panels depict TGIF2 single channel image of the area in the dashed box. Embryos are presented in lateral view. Scale bars, 50 μm. ht, heart; vpa, ventral pancreas. (**c**) Left, whole-mount *in situ* hybridisation analysis of *Tgif2* in 2S-stage mouse embryo. Embryo is presented in ventral view; arrow indicates lateral domains of the ventral fg. Right, *in situ* hybridisation on E12.5 mouse cryosections detects expression of *Tgif2* in the whole pancreatic epithelium (demarcated by yellow dotted line). Scale bar, 50 μm. pa, pancreas; st, stomach. (**d**) Schematic showing directed differentiation of mESC cultures into DE and, subsequently, pancreatic (PE) or hepatic endoderm (HE). On day (d) 8 of differentiation, PE and HE populations were analysed by RT-qPCR for the expression of the indicated genes in the presence or absence of LV-TGIF2. Induction of endogenous *Tgif2* transcript was observed during directed differentiation of ESCs toward endoderm, consistent with its expression profile in the gastrulating mouse embryo (**b**). Data were normalized to that of *Sdha* and shown as Log2-expression ratio relative to control undifferentiated ES cultures. Serp., Serpina1. Values shown are mean±s.e.m. (*n*=3). *P<0.05, **P<0.01 obtained with REST randomization test^63^ for differentiated PE or HE versus undifferentiated ESC; ^*#*^*P*<0.05, ^*##*^*P*<0.01 obtained for PE or HE+LV-TGIF2 versus non-transduced PE or HE.

The TALE class of homeodomain-containing TFs are known to play crucial roles in establishing cell identity and organogenesis, including pancreas formation^28^,^29^,^34^. We found that foregut endoderm progenitors express elevated *Tgif2* levels, which is in line and validated previous RNASeq data^25^ (Fig. 1a; Supplementary Fig. 1a). Importantly, at the 2-somite (S) stage (E8.0) *Tgif2* expression was spatially confined to the caudo-lateral region of the ventral foregut, which is the location of presumptive bipotent hepatic and pancreas progenitors (Fig. 1c)^35^. Subsequently, by 7–9S stage (E8.5), whole-mount immunofluorescence (IF) showed co-localization of TGIF2 with PROX1 in ventral pancreatic progenitors at the lip of the foregut but not in hepatoblasts (Fig. 1b). After the fate decision between liver and pancreas is made, *Tgif2* exhibited high and persistent expression levels in pancreas throughout embryonic development, as well as in adulthood, whereas it was undetectable in the liver (Fig. 1; Supplementary Fig. 1b,c).

**Figure 2:**
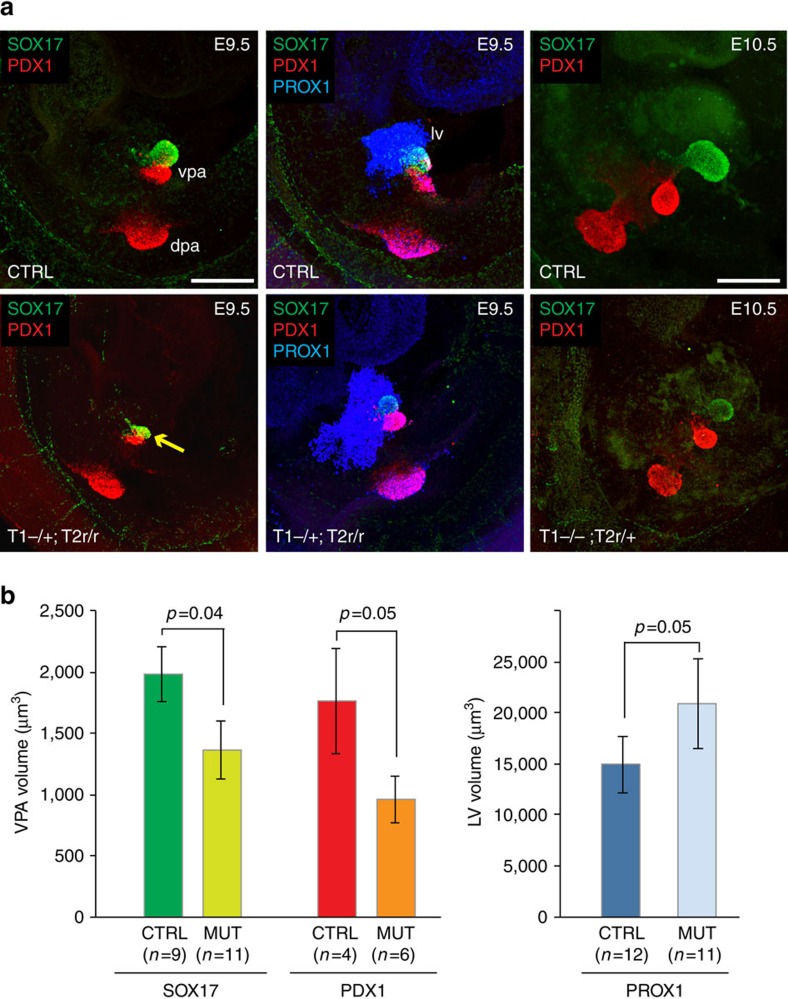
The TALE *Tgif* gene family controls lineage segregation within the ventral foregut endoderm of the mouse embryo. (**a**) Representative maximum confocal z-projections of PDX1/SOX17 and PDX1/SOX17/PROX1 IF analyses in control (CTRL), *Tgif1*^*−/−*^*;Tgif2*^*lox/+*^;*Sox2-Cre*^*+*^ (referred to as T1−/−; T2r/+) and *Tgif1*^*+/−*^*;Tgif2*^*lox/lox*^;*Sox2-Cre*^*+*^ (referred to as T1+/−; T2r/r) mouse embryos. PDX1 (red) marked both dorsal and ventral pancreatic buds and at E9.5 co-localized with SOX17 (green) in the pancreatobiliary progenitor population that gives rise to the ventral pancreas, extrahepatic ducts, and gall bladder (arrow). PROX1 (blue) marked both liver and pancreatic buds. dpa, dorsal pancreas; lv, liver; r, recombined; vpa, ventral pancreas. Scale bars, 50 μm. (**b**) Quantification of the ventral pancreas and liver volume buds from confocal images of E9.5 embryos. Average fluorescence intensity of PROX1^+^ liver buds, SOX17^+^ and PDX1^+^ ventral pancreas buds was measured using the ‘object analysis' function in Huygens software. Error bars indicate±s.e.m. *n*, indicated for each genotype. Statistics by two-tailed *t*-test and *P* values are shown.

Because of its restricted expression in the ventral foregut, we asked whether *Tgif2* regulates the allocation of foregut progenitor cells into pancreas fate and is sufficient for the activation of pancreatic transcriptional program. The closely related family member, *Tgif1*, does not display a similar restricted expression, being in both hepatic and pancreatic progenitor cells^25^. However, given the high degree of functional overlap between *Tgif2* and *Tgif1* during mouse embryogenesis^29^,^33^, we conditionally deleted *Tgif2* in the epiblast using a loxP-flanked *Tgif2* allele^36^ and the *Sox2*-Cre transgene^37^ in a *Tgif1*-deficient background^32^ (Fig. 2; Supplementary Fig. 1d). No double homozygous mutant embryos (Tgif2r/r;Tgif1−/−;Sox2-Cre) were found in our genetic background (0 out of 216 embryos), hampering a complete loss-of-function analysis (Supplementary Fig. 1d). However, the presence of one single wild-type allele of either *Tgif1* or *Tgif2* gene supported normal embryonic development to at least E10.5, enabling the analysis of ventral foregut organogenesis (Fig. 2). We analysed the expression of *Pdx1* and *Sox17*, which is involved in the segregation between liver and pancreatobiliary systems and expressed in ventral pancreatic progenitors from E9.5 onward^38^,^39^,^40^,^41^. Interestingly, mouse embryos with *Tgif* combined mutations, homozygous for one gene and heterozygous for the other (for example, Tgif2r/+;Tgif1−/−;Sox2-Cre or Tgif2r/r;Tgif1+/−;Sox2-Cre), showed consistent reduction of the SOX17^+^ and PDX1^+^ ventral pancreatic buds at E9.5 (Fig. 2a,b). This was accompanied by an expansion of the hepatic bud volume, as measured by PROX1 staining (Fig. 2a,b).

**Figure 3:**
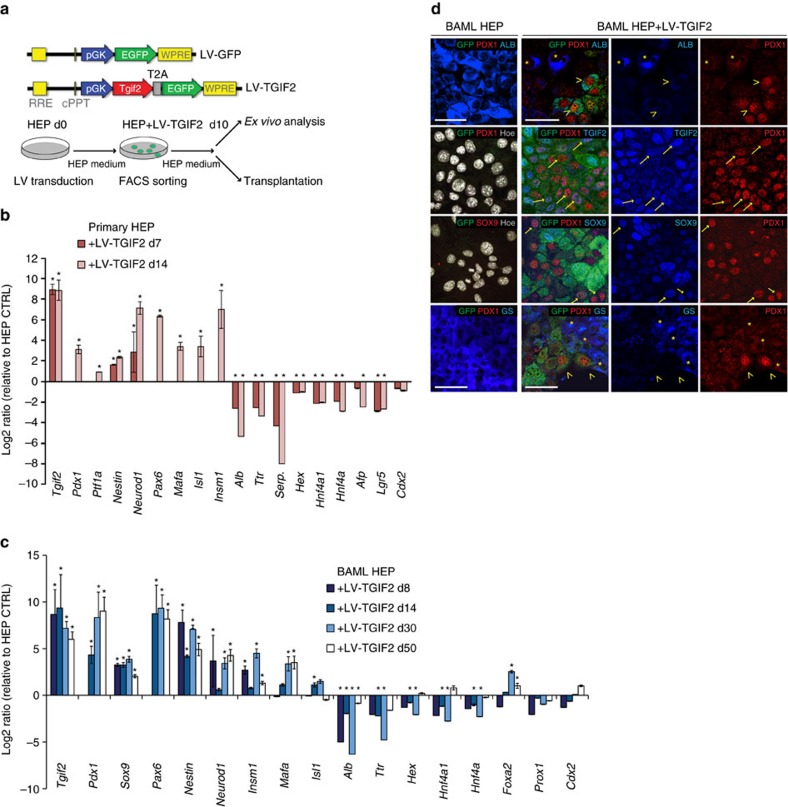
Tgif2 expression induces adult murine liver cells to acquire molecular features of pancreatic progenitors. (**a**) Map of LVs and schematic of the *ex vivo* reprogramming. Murine adult primary or BAML hepatocytes (HEP) were transduced with the constitutively active PGK-TGIF2-EGFP LV (LV-TGIF2) expressing *Tgif2* and EGFP, FACS-sorted for EGFP and characterized at different time points by various *ex vivo* and *in vivo* approaches. (**b**) RT-qPCR analysis of hepatic and pancreatic gene expression in mouse primary HEPs at d7 and d14 after transduction with LV-TGIF2. Data were normalized to *Sdha* and represented as Log2-expression ratio between LV-TGIF2-transduced and control hepatocytes at matched time-points. (**c**) RT-qPCR analysis of hepatic and pancreatic gene expression in murine adult BAML HEP cells transduced with LV-TGIF2 at the indicated time points after transduction. Data were normalized to *Sdha* and represented as Log2-expression ratio between LV-TGIF2-transduced and control cells. Values shown are mean±s.e.m. (*n*=5) *P<0.05. (**d**) IF of unsorted (no FACS) LV-TGIF2-transduced (right) BAML HEP cells and control cells (left). In top panel of LV-TGIF2-transduced cells, asterisks (*) indicate Albumin (ALB)-positive cells that are PDX1-negative; open arrowheads (>) indicate PDX1/GFP-positive cells that are ALB-negative in transduced cultures. In middle panels, arrows indicate examples of GFP-positive cells either PDX/SOX9-double or PDX1/TGIF2-double positive cells. In bottom panel, asterisks (*) indicate Glutamine synthetase (GS)-positive cells (blue) that are negative for PDX1 (red) in cultures d50 after transduction; open arrowheads (>) indicate PDX1-positive cells that are negative for GS. Scale bars, 50 μm.

Next, we directly assayed *Tgif2* for pancreatic fate-inducing activity. To model *ex vivo* endoderm development and specification of pancreas progenitors, we used a directed stepwise differentiation system of mouse embryonic stem cells (ESC), adapted from previously published studies^25^,^42^,^43^. Monolayer cultures of mES cells were first differentiated into definitive endoderm (DE), transduced with a bicistronic lentiviral vector (LV) expressing *Tgif2* and the reporter enhanced green fluorescent protein (EGFP) genes, and then differentiated into pancreatic endoderm (PE) (Fig. 1d; Fig. 3a). We found that *Tgif2* enhanced differentiation of the DE-induced population toward pancreatic fate, as judged by the further increase in expression levels of *Sox17, Pdx1* and *Hnf1b* (Fig. 1d). Conversely, when DE cells were differentiated into hepatic endoderm (HE)^44^ in the presence of *Tgif2*, we observed marked decrease in the expression of liver-specific genes (for example, *Serpina1*, *Hnf4a*, *C/EBPa*) (Fig. 1d). Taken together, our results indicate that TGIF2 acts as a developmental regulator of the hepatopancreatic lineage, pushing foregut progenitors toward pancreatic cell fate and counteracting hepatic fate. Thus, these properties make TGIF2 a pertinent candidate to be tested for lineage reprogramming of liver to pancreas.

### Stepwise reprogramming of liver cells to pancreas progenitors

To assess this possibility, first we developed an *ex vivo* lineage reprogramming strategy based on LV vectors for stable and sustained expression of *Tgif2* in liver cells. We expressed *Tgif2* in primary mouse hepatocytes (HEP) that were freshly isolated from adult livers and cultured in standard hepatocyte culture medium (Fig. 3a; Supplementary Fig. 2e). At one week after transduction with LV-TGIF2, we measured robust ectopic *Tgif2* expression and concomitant repression of liver-specific genes, including *Albumin, Serpina1* and *Transthyretin* (*Ttr*), and the TFs *Hex*, *Hnf4a* and *Hnf4a1* liver-specific isoform (Fig. 3b). Two weeks post-transduction, the depletion of hepatic transcripts was maintained (Fig. 3b) and accompanied by induction of pancreatic progenitor genes, such as the TFs *Pdx1, Ptf1a, Neurod1, Pax6, MafA, Isl1, and Insm1* (ref. 23), as well as *Nestin*, encoding an intermediate filament protein, which marks undifferentiated pancreatic precursor cells at E10.5 (ref. 45) (Fig. 3b; Supplementary Fig. 2f). Notably, the expression of all these pancreatic TFs, besides *Pdx1* and *Ptf1a*, begins at the onset of pancreatic bud formation by E10.5 and later is restricted to the endocrine cell population^23^,^25^. LV-TGIF2 did not affect the expression levels of endogenous *Tgif2* or *Tgif1* (Supplementary Fig. 2g).

Prolonged analysis of the LV-TGIF2-transduced cells beyond 2 weeks was not possible because of the limited viability of primary HEP cultures *in vitro*^46^,^47^. To overcome this problem, we used the well-characterized non-transformed BAML hepatic cell line^48^, as an additional *ex vivo* model system. The BAML is a cell line established from adult wild-type mouse liver that was previously shown to repopulate the liver *in vivo*^48^. This cell line displays various mature hepatic features, including expression levels of *Albumin, Serpina1*, *Ttr*, *Fah,* which are comparable to those in the adult mouse liver (Supplementary Fig. 2a). However, the level of important enzymes in glucose homoeostasis, such as *G6pc* and *Pepck,* and members of the cytochrome P450 family, such as *Cyp3a11*, is lower than in adult mouse liver, while biliary epithelial markers Cytokeratin 7 and 19, are present in BAML, like in bi-potential hepatic progenitor cells (Supplementary Fig. 2a). We cultured the BAML hepatic cells in their standard hepatocyte culture medium and characterized them after transduction with either LV-TGIF2 or LV-GFP, as control, at multiple time points *ex vivo*, as well as *in vivo* (Fig. 3c; Supplementary Fig. 2b). *Tgif2*-expressing cells proliferated at a lower rate than controls during the first week after transduction and reacquired the normal proliferative activity after 10 days (Supplementary Fig. 2c,d). At early time points (1 and 2 weeks), ectopic *Tgif2* expression in BAML HEP cells elicited effects similar to those observed in primary HEPs, including strong reduction of the hepatic gene expression program and subsequent induction of a set of pancreatic genes (Fig. 3c). Importantly, the levels of pancreatic transcripts either remained stably induced or increased (for example, *Pdx1* and *MafA*) over time (day (d) 30 and d50 time points after *Tgif2* expression) (Fig. 3c). Repression of the hepatic gene expression program was also maintained in long-term cultures of LV-TGIF2-tranduced BAML HEP cells, even though it became less prominent after d50, suggesting possible occurrence of culture heterogeneity (Fig. 3c).

IF analysis corroborated these findings, showing LV-TGIF2-transduced BAML HEP cells positive for GFP that were also positive for SOX9 and/or PDX1 but displayed reduction or loss of hepatic differentiation markers (albumin and glutamine synthetase (GS)) (Fig. 3d). Three weeks after transduction, a typical TGIF2-LV culture was composed of 50±4% SOX9-positive cells, 8.75±1.23% PDX1-positive cells and 48.10%±3.84% of the PDX1-positive cells also co-express SOX9. Notably, PDX1-positive cells were not simultaneously positive for liver markers, such as albumin or GS, excluding hybrid cellular phenotypes in LV-TGIF2 HEP cultures (Fig. 3d). As expected, control BAML HEP cultures displayed typical abundant cytoplasmic albumin or GS in the absence of pancreatic markers (Fig. 3d).

TFs in common between the two fates, such as *Prox1* and *Foxa2*, were expressed at similar levels in both control and *Tgif2*-expressing HEP cells (Fig. 3c). Induction of marker genes for stomach, duodenal and intestinal fates (for example, *Sox2, Nkx6.3* and *Cdx2*) was not detected in *Tgif2*-expressing HEP cells (Fig. 3b,c; Supplementary Fig. 3a,b). Moreover, induction of pancreatic fate was not accompanied by the activation of a facultative liver stem/progenitor population (for example, *Lgr5*-positive)^49^, pluripotency markers or other lineage-specific transcripts, suggesting a fate-restricted switch (Fig. 3b; Supplementary Fig. 3). Remarkably, we also observed induction of endodermal genes, such as *Gata6* and *Cxcr4*, as well as of *Pdx1* and *Ptf1a* expression when LV-TGIF2 was transduced in adult mouse fibroblasts, suggesting a potential broader reprogramming activity of TGIF2 also in non-endodermal lineage cells (Supplementary Fig. 3c).

**Figure 4:**
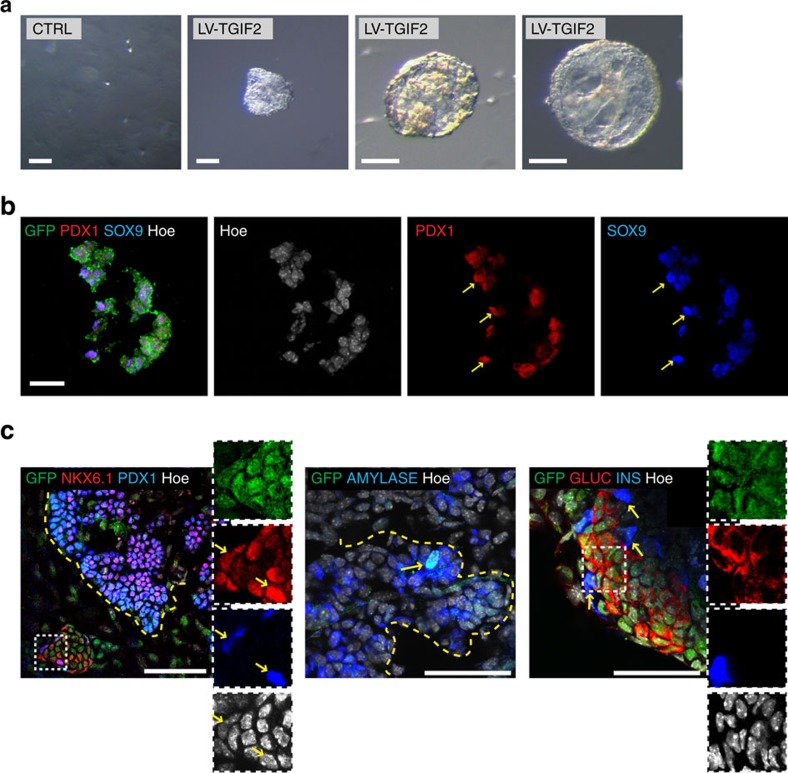
Primary hepatocytes transduced with LV-TGIF2 formed pancreatic organoid structures. (**a**) Micrographs of primary HEPs grown in three-dimension (3D) culture conditions for E10.5 mouse pancreas progenitor cells. Control (CTRL) primary hepatocytes failed to grow in the same conditions. All organoid structures shown in the figure were generated from LV-TGIF2-transduced primary hepatocytes. Bar, 100 μm. (**b**) IF staining on cryosections of LV-TGIF2-organoids for GFP (green), SOX9 (blue) and PDX1 (red). Hoechst (Hoe) nuclear counterstain in grey. Scale bar, 20 μm. (**c**) Whole-mount IF staining of pancreatic explants transplanted with LV-TGIF2-organoids. Insets are magnifications of the areas in the white boxes and shown as single channel images. Left panel, arrows indicate GFP-positive grafted cells that are positive for PDX1 and NKX6.1 (*n*=6, IF exp.). Middle panel, arrow indicates GFP-positive cell that is amylase-positive (*n*=5, IF exp.). Right panel, arrows indicate GFP-positive grafted cells expressing endocrine markers, insulin or glucagon (*n*=6, IF exp.). Dashed yellow line marks pancreatic epithelium. Scale bar, 50 μm.

Altogether these data show that *Tgif2* is uniquely able to rapidly down-regulate the typical hepatic gene regulatory network and promote a pancreatic progenitor gene-expression signature even in conditions that favour hepatic cell maintenance. We then tested if LV-TGIF2-transduced primary HEPs grow and expand in three-dimension (3D) culture conditions that have been recently established for pancreas progenitor cells^50^. We found that TGIF2-LV primary HEPs formed organoids, which were composed of SOX9- and PDX1-positive cells (Fig. 4a,b). By contrast, control non-transduced primary HEPs did not cluster to form 3D aggregates under the same pancreatic conditions and failed to grow (Fig. 4a). Next, to start testing the differentiation potentials of the organoids established from TGIF2-LV primary HEPs, we transplanted them into fresh E12.5 mouse pancreatic explants (Fig. 4c). Importantly, when exposed to their native pancreatic microenvironment, the grafted progenitor cells (GFP-positive) either expressed pancreatic progenitor markers (PDX1, NKX6.1) or retained the potential to differentiate and expressed acinar (AMYLASE) or endocrine (INSULIN, GLUCAGON) markers (Fig. 4c). Endocrine differentiation of GFP-positive grafted progenitor cells was more frequent into glucagon^+^ cells, resulting in average of ∼30% GLUC^+^/GFP^+^ versus 10% INS^+^/GFP^+^, while exocrine cells were less frequent (Fig. 4c). Altogether, these results indicate that in the presence of TGIF2 mature hepatocytes acquire a typical pancreatic progenitor gene expression signature and can further differentiate as true progenitor cells. Reprogrammed primary and BAML HEP cells are hereafter referred to as TGIF2-induced Pancreatic Progenitor (TiPP) cells.

### Tgif2-reprogramming induces gene expression remodelling

**Figure 5:**
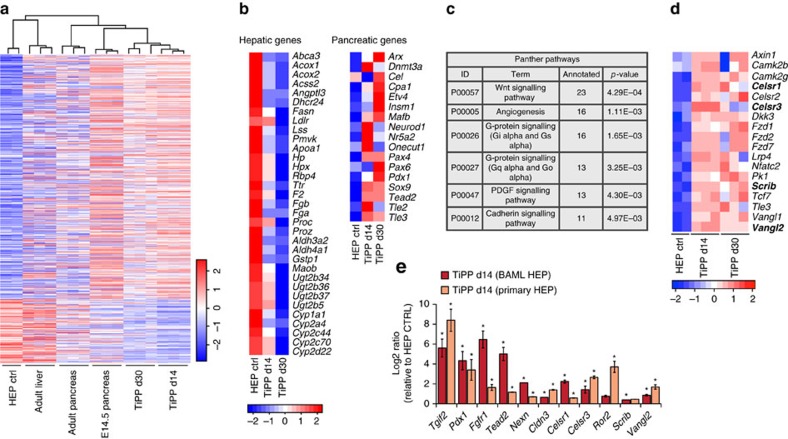
Lineage conversion is accompanied by global transcriptional remodelling. (**a**) Hierarchical clustering of the gene expression values of the sub-set of genes that were differentially expressed between BAML HEP control and TiPP cells at d14, derived from BAML HEPs+LV-TGIF2 (total 592 genes; *P*<0.01 and FC >4 or <−4) among all samples analysed. Gene probes are located in rows and samples in columns. Gene expression across rows in the heatmap are coloured according to the z-score, such that the mean expression of each row is set to white colour and expression values higher or lower than the mean are graded towards red or blue, respectively. Rows in the heatmap are ordered according to the FC. (**b**) Heat map (left) illustrating the relative expression levels of selected hepatic genes involved in metabolic activities in HEP control and TiPP cells. Heat map (right) illustrating the relative expression levels of selected pancreatic progenitor genes in HEP control and TiPP cells. The colours in the heatmap are normalized per row by calculating the z-score for each row. Replicates were summarized as the mean. (**c**) Panther pathways enrichment analysis of differentially regulated genes in HEP control and TiPP cells at d14 (*P*<0.05 and FC >2 or <−2). For complete gene ontology annotation lists see Supplementary Data 1. (**d**) Heat map illustrating the relative expression levels of selected Wnt signaling pathway genes. Genes in bold were selected for confirmation by RT-qPCR. (**e**) Confirmation of microarray gene expression changes by RT-qPCR analysis. Data were normalized to *Sdha* and represented as Log2-expression ratio between TiPP d14 cells derived from either BAML HEP or primary HEP and their respective control cells. Values shown are mean±s.e.m. (*n*=3) *P<0.05.

Next, we analysed the changes in global gene expression in reprogrammed TiPP cells. We compared the gene expression profiles of FACS-purified TiPP cells at two time points (d14 and d30 post-transduction), control BAML HEP cells, mouse embryonic pancreas (E14.5) and adult pancreas and liver tissues. Unsupervised hierarchical clustering of gene expression data showed a clear relationship between reprogrammed TiPP cells and E14.5 mouse pancreas (Fig. 5a). As expected, the control BAML HEP population clustered together with adult liver (Fig. 5a). Notably, typical representative genes, which define pancreatic progenitor identity, including *Sox9*, *Pdx1*, *Pax6*, *Cpa1*, *Nr5a2*, *Neurod1*, *Tead2, Tle2*, *Tle3*, were upregulated in TiPP cells, whereas all well-known genes involved in hepatic functions, such as cytochrome enzymes, apolipoproteins and detoxification enzymes, were down-regulated (Fig. 5b).

Embryonic pancreas and liver share many common features, which is reflected by the similarity of their transcriptome profiles^24^ (Supplementary Fig. 4). To measure to what extent TiPP cells are closer to pancreatic or hepatic progenitors, we first focused on the initial events of reprogramming (at d14 time point) and examined the overlap between the gene expression-signature of mouse E10.5 FACS-purified pancreatic and hepatic progenitors^25^, TiPP d14 and control population transcriptomes (Supplementary Fig. 4a,b). We found that more than 50% of the pancreas progenitor genes were upregulated in TiPP d14 cells, indicating a significant overlap (FC>2, *P* value <0.05, Benjamini–Hochberg method) despite the methodological differences (Supplementary Fig. 4a). By contrast, only 4% of E10.5 hepatic genes were upregulated in TiPP cells (FC>2, *P* value <0.05) (Supplementary Fig. 4b). Similarly, the comparison of the expression profiles of reprogrammed cells with mouse embryonic liver and pancreas at later stages (E13.5–E14.5) showed that TiPP cells gained a larger fraction of pancreatic-specific markers compared with hepatoblast-specific markers (Supplementary Fig. 4c,d). Thus, the reprogramming event was broadly reflected in global gene expression changes, suggesting that TiPP cells are similar to mouse pancreatic progenitors.

Analysis of gene ontology terms enrichment of differentially regulated TiPP genes revealed an upregulation for genes involved in developmental processes, cell adhesion and transcription, while genes involved in metabolic and transport processes were downregulated (Supplementary Fig. 4f; Supplementary Data 1). This is in line with the robust inhibition of typical hepatocyte functions (Fig. 5b). Interestingly, the analysis of enrichment of Panther pathway annotations pointed to genes involved in the Wnt signalling pathway (23 genes; *P* value, 0.00043) (Fig. 5c). In particular, we found that many Wnt/Planar cell polarity core genes, such as *Vangl*, *Celsr*, *Prickle1* and *Scrib*, as well as downstream Wnt transducers, such as *Tcf7, Nfatc2*, *Camk2b* and *Tle3,* were upregulated in reprogrammed cells (Fig. 5d,e). These findings are consistent with the previously recognized role for non-canonical Wnt in pancreas and liver cell lineage divergence and imply concurrent changes in epithelial cell polarity during liver-to-pancreas fate conversion, as seen in the embryo^25^,^51^. Finally, since TGIF2 is a known regulator of TGF-β and BMP signalings^28^,^33^, we searched for potential modulation of their target genes in TiPP and control population transcriptomes. We observed a strong downregulation of well-known direct BMP targets (*Id1* and *Id2*) in TiPP cells, while the effect on TGF-β target genes is less prominent (Supplementary Fig. 4e). These findings are consistent with previous reports on TGIF transcriptional regulation of BMP/TGF-β signalling^28^,^33^ and the role of BMP in pancreas versus liver fate decision^24^.

### Induced pancreatic progenitors differentiate *in vivo*

**Figure 6:**
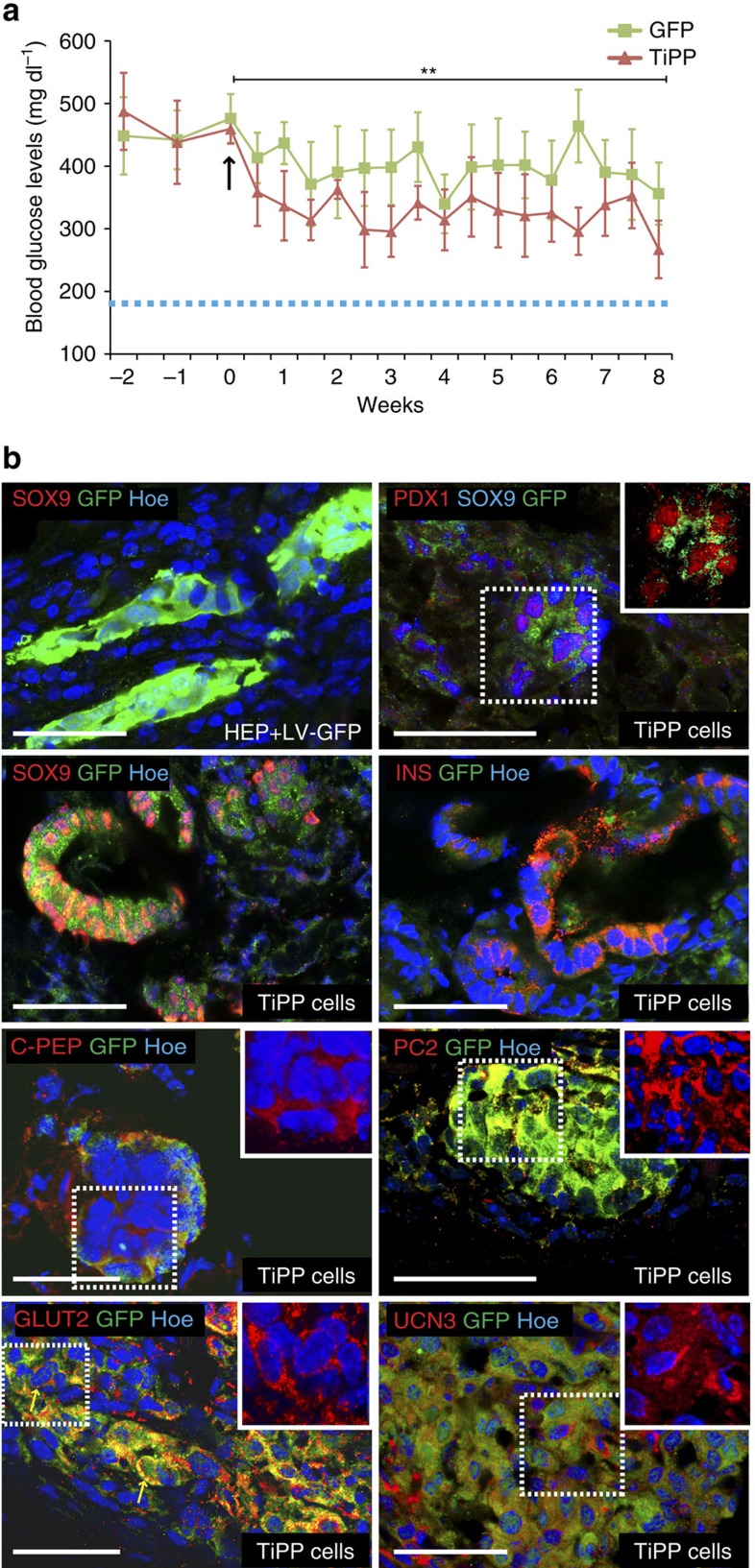
*In vivo* analysis of TGIF2 reprogramming activity. (**a**) Transplantation assays in Akita diabetic mice. Reprogrammed TiPP cells (derived from BAML HEPs+LV-TGIF2 d8 post-transduction) were grafted under the KC of hyperglycemic Akita mice (red line). Animals engrafted with BAML HEP cells transduced with LV-GFP were used as negative controls (green line). Blood glucose levels were measured under non-fasting conditions before transplantation (time points: −2 wk and −1 wk), the day of transplantation (time point 0; arrow) and, subsequently, twice a week after transplantation. *n*=6 animals in each group. Normoglycemia is defined as blood glucose level below 200 mg dl^−1^ in wild-type littermate mice under nonfasting conditions (dotted line). Values shown are mean±s.e.m. **P<0.01 (two-way ANOVA comparison test between LV-GFP and TiPP transplanted groups). (**b**) IF analysis on cryosections of mouse KCs transplanted with LV-GFP BAML HEP cells and TiPP cells. Hoechst (Hoe) nuclear counterstain in blue. Insets show area in the dashed box of: PDX1 (red)/GFP (green) staining without SOX9 channel (blue); C-peptide (C-PEP) (red)/Hoechst (blue) staining without GFP channel (green); PC2 (red)/Hoechst (blue) staining without GFP channel (green); UCN3 (red)/Hoechst (blue) staining without GFP channel (green); GLUT2 (red)/Hoechst (blue) staining without GFP channel (green), arrows indicate staining near the membrane, as expected for the location of mature Glut2 protein. To a lesser extent, some cells displayed Glut2 staining in the cytoplasm, which is similar to neonatal beta-cells. Scale bars, 50 μm.

A stringent test routinely used for pancreatic progenitor cells obtained *in vitro* is whether they ‘mature' to functional cells when implanted *in vivo*^42^,^43^. To assess the *in vivo* differentiation potential of the reprogrammed-derived pancreatic progenitors, we engrafted the TiPP cells (1 × 10^6^ cells) under the kidney capsule (KC) of hyperglycemic Akita mice. Islets from Akita heterozygous mice are depleted of β-cells, and those remaining release very little mature insulin, representing an excellent mouse model of diabetes^52^. We found that blood glucose levels improved soon after transplantation of TiPP cells and remained stable over the eight weeks duration of the experiment (Fig. 6a), displaying an effect comparable to the transplantation of 100–200 mouse islet equivalent (IE) (Supplementary Fig. 5). IF analysis revealed that TiPP cells implanted under the KC organized themselves into epithelial structures, which were positive for PDX1 and SOX9 or for typical molecular features of pancreatic β-cells, such as GLUT2, insulin, C-peptide, prohormone convertase (PC) 2 and urocortin 3 (UCN3) (Fig. 6b; Supplementary Fig. 5e). In particular, within the grafts ∼30% INS-positive cells were detected, (29±2%), reflecting the efficiency of differentation of TiPP cells upon engraftment. By contrast, neither amylase nor other endocrine hormones were detected in the grafted TiPP cells (Supplementary Fig. 5d). Akita mice transplanted with BAML HEP cells transduced with control LV-GFP displayed higher levels of glycaemia throughout the assay and did not acquire pancreatic differentiation features in the KC mesenchyme (Fig. 6a,b; Supplementary Fig. 5). No tumours were observed after eight weeks posttransplantation. These results indicate that pancreatic progenitors derived from reprogrammed hepatic cells are ‘poised' for further differentiation along the endocrine β-cell lineage when exposed to the appropriate environment. Nevertheless, the obtained insulin-producing cells are likely immature beta-like cells and do not fully recapitulate functional capacity of mature beta-cells.

### *In vivo* induction of pancreas progenitors

**Figure 7:**
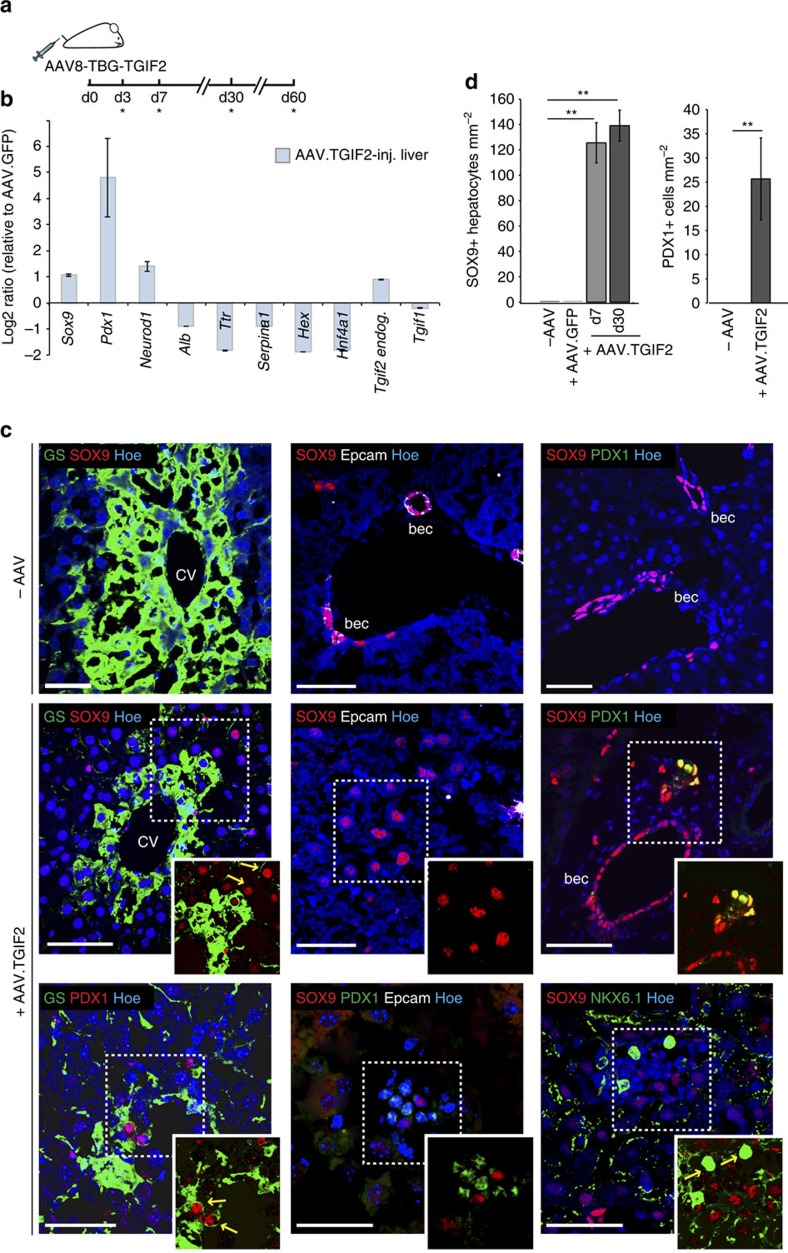
*In vivo* analysis of TGIF2 reprogramming activity. (**a**) Schematic of the AAV-mediated experimental strategy to express *Tgif2 in vivo* in the adult mouse liver. AAV-injected and uninjected control livers were examined at the indicated time points (*) after *iv* injection. (**b**) RT-qPCR analysis of indicated genes in AAV.TGIF2-injected livers at d30. Data were normalized to 36B4 and represented as Log2-expression ratio between AAV.TGIF2-injected and AAV.GFP-injected livers. Values shown are mean±s.e.m. (*n*=3). (**c**) IF staining of AAV-injected (+AAV.TGIF2) and uninjected (−AAV) adult mouse livers for GS, SOX9, Epcam, PDX1 and NKX6.1. Micrographs show IF stainings performed at d30 after AAV.TGIF2 injection; SOX9/NKX6.1 IF staining was done at d60. SOX9 and Epcam mark biliary epithelial cells (bec) and are absent in hepatocytes in uninjected livers. Arrows indicate examples of SOX9−, PDX1− or NKX6.1-positive hepatocytes in AAV.TGIF2-injected livers. Outlined areas are shown in the insets without Hoechst nuclear counterstain (blue). CV, central vein. Scale bars, 50 μm. (**d**) Quantification of SOX9-positive hepatocytes and PDX1-positive cells in adult livers injected with AAV.GFP (*n*=2 d30), AAV.TGIF2 (*n*=3 d7; *n*=4 d30) and uninjected (*n*=3). Average number of labelled cells per section (mm^2^) was determined for each animal in one entire liver lobe. Results are expressed as the mean±s.e.m. Statistics by two-tailed *t*-test. **P<0.01.

Given its remarkable *ex vivo* reprogramming activity, we next assessed whether TGIF2 might induce a pancreatic program *in vivo* directly in the native liver environment. To exclusively target hepatocytes, we ectopically expressed *Tgif2* using recombinant AAV2/8 vector, which exhibits hepatocyte-specific tropism and rapid clearance, and combined it with the hepatocyte-specific thyroid-binding globulin (TBG) promoter^53^ (Fig. 7a). Livers from adult animals administered with AAV8.TBG-Tgif2 were collected at different time points after delivery and examined for cellular identity by RT-qPCR and IF analyses (Fig. 7a–c; Supplementary Figs 6–8). Normal tissue-architecture was preserved in AAV-injected livers, as judged by their lobular organization along the porto-central axis and distinct periportal and perivenous gene expression patterns (Fig. 7c; Supplementary Figs 6 and 7). Notably, within one week of AAV8.TBG-Tgif2 injection, we detected ectopic SOX9-positive cells throughout the liver parenchyma (Fig. 7c,d), including in the perivenous ‘zone 3' of the liver that is close to the central vein and positive for GS, whereas in adult livers under normal conditions SOX9 marks only biliary epithelial cells (bec)^26^,^49^,^54^,^55^ (Fig. 7c). Ectopic SOX9 activation in hepatocytes of AAV8.TBG-Tgif2-injected livers was not accompanied by expression of bec markers (for example, Epcam, Osteopontin and CK19)^47^,^53^,^54^, excluding the induction of biliary fate (Fig. 7c; Supplementary Fig. 7a). One month post-AAV injection, we observed induction of PDX1 expression in a subset (18%) of SOX9-positive cells as well as additional pancreatic TFs, such as NeuroD1 and Nkx6.1 (Fig. 7c,d), but neither endocrine nor exocrine differentiation functions (Supplementary Fig. 7b) in the livers of AAV8.TBG-Tgif2-injected animals. AAV injection itself (for example, AAV.GFP) did not induce ectopic activation of pancreatic genes in hepatocytes (Fig. 7c,d; Supplementary Fig. 6c). Next, we asked whether environmental cues, such as a hyperglycemic state, might promote maturation of the Tgif2-induced pancreatic progenitor-like cells *in vivo* too. AAV8.TBG-Tgif2 was injected into diabetic Akita heterozygous mice, blood glucose levels and insulin concentration were measured at d60 after AAV delivery (Supplementary Fig. 6e,f). While blood glucose levels remained elevated in non-treated and AAV.GFP-injected Akita mice, a group of AAV.TGIF2-treated Akita animals (4 out 7) showed sustained decrease in blood glucose, which was accompanied by higher levels of circulating insulin (Supplementary Fig. 6e,f). The others remained hyperglycemic with low insulin levels. Altogether, these results indicate that ectopically expressed TGIF2 exerts similar activity *in vivo*, being sufficient to induce typical pancreatic progenitor genes expression, during normal adult liver homoeostasis.

## Discussion

Our data show that TGIF2 functions as a versatile reprogramming factor, exerting a twofold activity: it unlocks and represses hepatic identity and establishes pancreatic cell identity. Hepatic cells undergoing fate switching pass through an intermediate state that lacks characteristics of both the initial and final cellular identities, a so-called dedifferentiation step. Importantly, we found that this intermediate state is not widely plastic and *Tgif2*-expressing cells are instead ‘lineage-restricted' toward the pancreatic progenitor fate, without reverting to pluripotency or switching to other closely related lineages. Moreover, given the fact that adult hepatocytes are differentiated and arrested in quiescence (G0 phase), the change in cell identity triggered by *Tgif2* occurs in the absence of cell division (Supplementary Fig. 8), possibly implying a direct fate transitioning through discrete steps.

Previous liver-to-pancreas reprogramming studies used adenovirus vectors to force the expression of pancreatic TFs (for example, Pdx1, Ngn3) in the liver *in vivo* or hepatic cell cultures^17^,^18^,^20^. These studies mostly focused on the acquisition of insulin expression and some other markers of pancreatic identity, but they did not assess the degree of loss of hepatic fate and did not follow the fate transition (liver to pancreas) temporally. Importantly, in the strategy presented here, we showed that TGIF2 first represses hepatic gene expression, which is followed by induction of pancreatic progenitor genes. Such temporal succession of the events is a prerequisite to avoid aberrant hybrid cellular states in lineage reprogramming. In addition, in previous studies the forced expression of pancreatic TFs seems to work only under certain conditions that might be favourable to cellular transdifferentiation^17^,^18^,^20^. For instance, adenovirus-mediated delivery of Pdx1 or Ngn3 was shown to induce insulin expression by the liver, whereas AAV-mediated overexpression of the same TFs did not result into insulin production or correction of diabetes in mice^19^. These observations suggest that adenoviruses may facilitate cellular reprogramming, possibly because of their ability to induce general inflammatory responses, which is instead absent upon AAV gene transfer^19^,^56^. It is therefore crucial to assess the exact contribution of the reprogramming factor(s) itself in the absence of an inflammatory microenvironment. For this reason, in our novel strategy we assessed the specific reprogramming activity of TGIF2 using independent *in vivo* and *ex vivo* approaches and different delivery methods, including AAV under liver homoeostasis.

TGIF2 has been characterized as a context-independent transcriptional repressor, being able to exert its function either through modulation of the BMP/TGF-beta pathway or direct DNA binding^28^,^29^,^31^. Interestingly, the transcriptome analysis of the TiPP cells shows a higher number of upregulated than downregulated genes (470 out of 592 differentially regulated genes; *P*<0.01, FC<−4 or >4). If TGIF2 acts solely as a repressor, one possible mechanism of action to achieve activation of so many genes would be through the repression of a repressor. Alternatively, TGIF2 may function simultaneously as both an activator and repressor of genes during reprogramming. This is indeed a common feature of other TALE homeoproteins to act both as transcriptional repressor or activator in a context-dependent manner, for example, depending on cellular environment and binding partners^29^,^30^. Systematic characterization of TGIF2-mediated transcriptional activity will establish if this is the case also for this homeoprotein.

Reprogramming of hepatic cells to a pancreatic identity presents a valuable model to study hepatopancreatic plasticity and to identify by which mechanism each fate is established and maintained. Consistently, we identified a subset of molecular networks that are activated both in TGIF2-induced reprogramming as well as during liver and pancreas fate decision in the embryo. For instance, the enrichment of numerous non-canonical Wnt players in TiPP cells is consistent with the RNA-Seq profiling of mouse pancreatic and hepatic progenitor cells at the time of their lineage divergence^25^. In addition to Wnt modulation, we found robust and stable upregulation of Fgfr1 and some of its well-known downstream signal transducers, including Akt3, Plcg2, Mapk12, in the reprogrammed TiPP cells when compared with control hepatocytes (Supplementary Table 1). Recent observations identified a regulatory loop between SOX9 and FGF signalling in the early pancreatic *niche* that is required to repress liver-specific gene expression program and ensure pancreatic fate identity at early time point^57^. Thus, our findings suggest that TGIF2 may install a similar circuit between SOX9 and Fgfr in hepatocytes that undergo reprogramming, recapitulating the same *in vivo* mechanisms. Notably, we also found induction of the transcription factor Tead2 in TiPP cells at early stage of reprogramming. This is in in line with the Teads being pancreatic progenitor-enriched factors in the mouse embryo^25^ and human pancreatic progenitors^58^, where they act as integral components of the pancreatic lineage-specific transcriptional program, regulating SOX9 and FGF receptors^58^.

From a broader perspective, the reported TGIF2-mediated stepwise fate conversion affords a unique opportunity to decipher genetic and epigenetic changes during the intermediate states of liver-to-pancreas reprogramming. This might also shed light on more fundamental reprogramming mechanisms. Indeed, fate conversion via a ‘tissue-restricted' dedifferentiation step, which precedes re-differentiation into the new cell type, has been reported in natural transdifferentiation events in worms^59^, as well as during limb regeneration in the axolotl (ref. 60), suggesting possible common themes in reprogramming and regeneration across phyla. The knowledge gained from these experimental manipulations will have broad implications not only for understanding the molecular basis of cellular identity and plasticity, but also for potential clinical application. Ultimately, the stepwise TGIF2-dependent fate conversion represents a novel strategy for controlled generation of pancreatic progenitors from liver and, possibly, a starting point for future production of pancreatic β-cells suitable for therapeutic use in diabetic patients.

## Methods

### Cell culture and viral infection

Mouse ES cells (R1 line)^61^ were maintained on gelatin-coated plates with inactivated mouse embryonic fibroblasts (MEFs) in standard mESC medium (High Glucose DMEM (Invitrogen) supplemented with 15% Fetal Calf Serum (FCS) Gold Serum (PAA), non-essential amino acids (Invitrogen), 1% Penicillin/Streptomycin (Invitrogen), 10 mM sodium pyruvate (Invitrogen), 1% Glutamax (Invitrogen), 50 μm 2-mercaptoethanol (Invitrogen)) and 1,000 U ml^−1^ leukaemia inhibitory factor (ESGRO). For differentiation, cultures were MEF-depleted and seeded in mESC medium at high-confluence on gelatin-coated dishes. Monolayer differentiation was carried out as following^42^,^43^: DE medium to day 2 consisted of RPMI medium (Invitrogen) and 0.2% FCS supplemented with 50 ng ml^−1^ Activin A and 25 ng ml^−1^ Wnt3a at day 1 and Activin A only at day 2. PE medium to day 8 consisted of RPMI medium and 2% FBS supplemented with 3 ng ml^−1^ Wnt3a and 50 ng ml^−1^ FGF10 (refs 42, 43). HE medium to day 8 consisted of RPMI medium and 2% FBS supplemented with 1% B27, 10 ng ml^−1^ basic FGF and 20 ng ml^−1^ BMP4 (ref. 44). All recombinant proteins were purchased from R&D System unless otherwise stated. Plateable male mouse CD-1 cryopreserved primary hepatocytes (Life Technologies) and BAML cells were cultured on Collagen I-coated plates in hepatocyte medium^48^ (referred to as ‘HEP medium'), which consists of William's E medium without phenol red (Sigma), 1% Penicillin/Streptomycin, 1% Glutamax, 10% FCS, 30 ng ml^−1^ IGF II (Peprotech), 50 ng ml^−1^ EGF (R&D), 10 μg ml^−1^ Insulin (Peprotech), 0.1 μm dexamethasone and 10 mM nicotinamide. Mouse adult fibroblast cultured were established from tail biopsies and cultured in DMEM supplemented with 10% FCS. Cell lines were routinely tested for normal karyotype and mycoplasma. Cells were infected with pPGK-TGIF2-2A-EGFP lentiviral expression vector (LV-TGIF2) at multiplicity of infection (MOI) of 40. The pPGK-TGIF2-2A-EGFP lentiviral expression vector (LV-TGIF2) was generated by cloning the cDNAs of mouse Tgif2 and EGFP, linked by the T2A self-cleaving sequence, into the pRRL.SIN.cPPT.PGK-GFP.WPRE lentiviral plasmid vector (Addgene plasmid 12252).

### FACS experiments

For FACS isolation, hepatic cell suspensions were first filtered through a BD Falcon tube with cell strainer cap (BD 352235). Before sorting, propidium iodide (PI) was added to exclusively select live cells. After dead cell exclusion (SSC-A/PI-A), GFP-expressing cells were sorted using a FACS Aria I flow cytometer (BD Biosciences) using a GFP filter and setting the gate on the GFP fluorescence intensity. For cell cycle analysis, cells were fixed in 70% ethanol overnight and incubated with 100 μg ml^−1^ RNase A and 40 μg ml^−1^ PI for 30 min at 37 °C. The cell cycle profile was analysed using the BD LSRFortessa cell analyser. For generation of the growth curve, cells were seeded on collagen-coated plates on day 12 post-transduction at a density of 2.5 × 10^4^ cells per well. The medium was changed daily, and the cell number was counted every day for one week.

### 3D culture and transplantation assay in explants

For 3D-differentiation^50^ control primary hepatocytes or after transduction with LV-TGIF2 were dissociated and about 8–10 cells per μl mixed with Matrigel (BD Biosciences) at a ratio 1:3 and plated in pancreas progenitors culture conditions^50^. For transplantation assay into pancreatic explants, LV-TGIF2-organoids after 6 days in culture were roughly dissociated in 0.05% trypsin and injected into the recipient explants with a mouth pipette. E12.5 pancreatic explants were isolated and cultured as described in ref. 62. After engraftment, the explants were cultured for 4 days, fixed in 4% paraformaldehyde and analysed by immunofluorescent staining. The experiment was repeated twice with a total of 28 TGIF2-organoids successfully grafted into recipient pancreatic explants^63^.

### Immunohistochemistry and *in situ* hybridisation

Mouse embryos and tissues were fixed in 4% paraformaldehyde at 4 °C from 2 h to overnight. For cryosectioning, samples were equilibrated in 20% sucrose solution and embedded in OCT compound (Sakura). Whole-mount *in situ* hybridisation and *in situ* hybridisation on cryostat sections (10 μm) were performed according to ref. 28. For whole-mount immunostainings fixed mouse embryos were bleached for 1–2 h in 6% hydrogen peroxide solution, incubated in freshly prepared PBSMT blocking solution (2% milk powder, 0,5% TritonX-100 in 1 × PBS), and afterwards with primary antibodies at the appropriate dilution ON at 4 °C (see Supplementary Table 2). After extensive washes in fresh PBSMT solution at least 5–8 times, the embryos were incubated overnight with secondary antibodies at 4 °C in PBSMT solution^25^,^64^. Whole mouse embryos from E 9.5 onward were cleared in methyl salicylate for confocal microscope imaging. For immunostaining, cryosections were incubated with TSA (Perkin-Elmer) blocking buffer for 1 h at room temperature and afterwards with primary antibodies at the appropriate dilution (Supplementary Table 2). If necessary, antigen retrieval was performed by boiling the slides for 20 min in citrate buffer (Dako). For IF on cultured cells, cells were grown on coverslips, fixed in 4% paraformaldehyde for 20 min at RT and permeabilised in 0.2% Triton X-100 in PBS. Images were acquired on Zeiss AxioObserver and Zeiss LSM 700 laser scanning microscope. Huygens software (SVI) was used for 3D volume measurement analysis of confocal z-stacks of mouse embryos. For quantification of SOX9− and PDX1-positive cells, one entire lobe of AAV-injected and uninjected livers were sectioned, sampled at a minimum of every 150 μm and processed by immunohistochemistry. Liver sections were acquired using tile scan (mosaic) function on a Zeiss LSM 700 laser scanning microscope, cells were counted from digitized micrographs and area (Hoechst-positive) measured using ImageJ or Axiovision softwares. Sections were counted from at least three animals per condition. Values presented are the average cell number relative to the area of liver section (mm^2^). Student's *t*-test was used for statistical analysis.

### RT-qPCR and transcriptome analysis

RNA was isolated using RNAzol (Biozol) or High Pure RNA Isolation Kit (Roche). Total RNA was processed for reverse transcription (RT) using Transcriptor First Strand cDNA Synthesis Kit (Roche). Real-time PCR reactions were carried out using the SYBR Green Master Mix (Roche) on ABI StepOne Plus system. Succinate dehydrogenase (SDHA) or 36B4 were used as reference genes. Primer sequences are provided in Supplementary Table 1. Technical triplicates were run for all samples and ‘no RT' and no template controls were included in all experiments. All the values were normalized to the reference genes and calculated using the software REST^63^. qPCRs were performed for 40 cycles. ‘Undetermined' data points were assigned a Ct of ‘40' to enable calculation of fold change. Also, reactions with Ct-values equal to the ‘no RT' control reaction were considered as ‘negative' and the values used for calculating fold changes. Statistical significance in qPCR experiments was calculated using the REST randomization test^63^. Whole transcriptome analysis was performed on Agilent SurePrint G3 Mouse GE 8 × 60 K Microarray Kits at the MPI Array Facility, Dresden. The single colour signals were read using Agilent Feature Extraction Software. All bioinformatics analyses were performed using R (http://www.r-project.org). Differential gene expression was determined using the R statistical package limma^65^. Expression values were scaled per row by Z-score (row average equals zero (white); positive or negative values indicate how many standard deviations the value is located above (red) or below (blue) the mean, respectively). Adjusted *P* values were corrected for multiple testing using the Benjamini–Hochberg method. Gene Ontology analysis was performed using the Genecodis application^66^.

### Mouse strains

C57BL/6-Ins2^Akita/^J (ref. 52), TGIF1^tm1aPah^ (ref. 51) and Tg(Sox2-cre)^1Amc/J^ (ref. 37) (JAX laboratories) mouse strains were previously described. The mouse TGIF2^tm1a(EUCOMM)Wtsi^ strain was generated at the ICS using the EUCOMM embryonic stem (ES) cell collection^36^. All animal experimentation was carried out in accordance to the local ethics committee for animal care.

### Transplantation underneath the kidney capsule

Spontaneously diabetic 8–9 weeks old C57BL/6-Ins2^Akita^/J female mice were used as recipients. 100–200 mouse IE or 1 × 10^6^ FACS-sorted cells were collected and transplanted under the kidney capsule, according to previously reported procedures^67^. Blood glucose was measured at 12 h intervals over the first two days post-transplantation and biweekly thereafter. After 8 weeks, the graft-bearing kidneys were removed and fixed overnight in 4% formalin at RT. Samples were embedded in paraffin for histological analysis.

### AAV experiments

The adeno-associated viral construct AAV8.TBG.TGIF2 was generated by cloning mouse *Tgif2* cDNA into the pENN.AAV2/8.TBG.PI.RBG plasmid vector (PennVector p1015). Replication-incompetent AAV8.TBG.TGIF2 and AAV8.TBG.eGFP AAVs were produced at the Penn Vector Core facility. 2–4 × 10^11^ particles of adeno-associated virus diluted in sterile PBS were administered per adult mouse (C57BL/6 and C57BL/6-Ins2^Akita^/J strains) by tail vein injection.

### Glucose tolerance test

Glucose tolerance test (GTT) was carried out on animals that had been fasted overnight, before the day of experimentation (time point −1). Animals were injected *i.p.* with glucose (2 g kg^−1^ body weight). Glucose levels were measured from blood collected from the tail immediately before the glucose challenge (time point 0) and 15, 30, 60, 120 and 240 min after the glucose injection using a blood glucose metre (Contour, Bayer). Serum insulin concentration was measured with the Ultra Sensitive Mouse Insulin ELISA kit (Crystal Chem. #90080).

### Statistical tests

All results are expressed as mean±s.e. (s.e.m.). Sample sizes of at least *n*=3 were used for statistical analyses except where indicated. The significance of differences between groups was evaluated by two-tailed unpaired Student's *t*-test or, when more than two groups were assessed, by ANOVA test. *P*<0.05 was considered statistically significant. Statististical tests relevant to the qPCR and microarray experiments are described in the ‘RT-qPCR and transcriptome analysis' section.

### Data availability

Gene expression data have been deposited in GEO (Gene Expression Omnibus) under accession code GSE54591. The authors declare that all other data supporting the findings of this study accompany the article and its Supplementary Information Files, or are available from the corresponding author on reasonable request.

## Additional information

**How to cite this article:** Cerdá-Esteban, N. *et al*. Stepwise reprogramming of liver cells to a pancreas progenitor state by the transcriptional regulator Tgif2. *Nat. Commun.* **8,** 14127 doi: 10.1038/ncomms14127 (2017).

**Publisher's note:** Springer Nature remains neutral with regard to jurisdictional claims in published maps and institutional affiliations.

## Supplementary Material

Supplementary Information Supplementary Figures, Supplementary Tables, Supplementary References.

Supplementary Data 1 List of Gene Ontology (GO) enrichment terms generated from the microarray datasets of TiPP reprogrammed cells and CTRL hepatic cells.
