# Supplementary material for: Stepwise reprogramming of liver cells to a pancreas progenitor state by the transcriptional regulator Tgif2
Source: Nat Commun. 2017 Feb 13;8:14127. doi: 10.1038/ncomms14127 (PMC5316826; doi:10.1038/ncomms14127)
Supplement: Supplementary Information — Supplementary Figures, Supplementary Tables, Supplementary References. [file ncomms14127-s1.pdf]

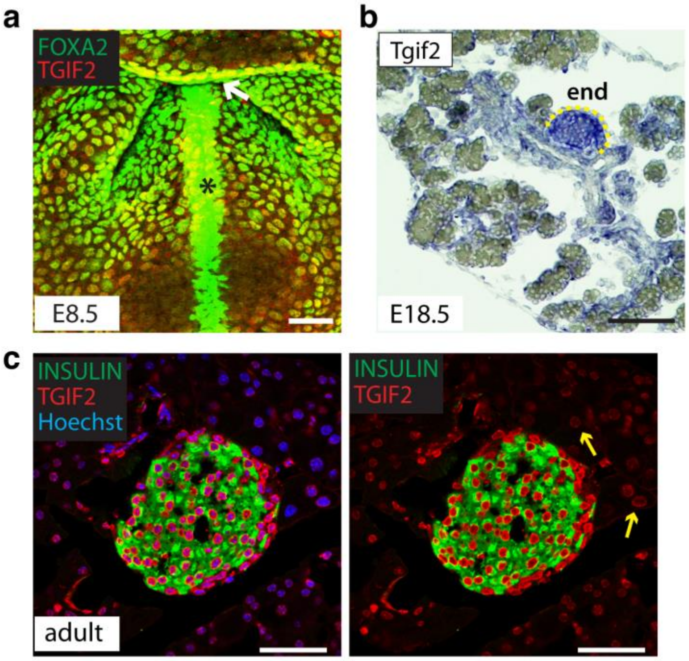

d

genotype	#	observed	expected
Cre+; TGIF1-/-; TGIF2 r/r	0	**0.00%**	3.13%
Cre+; TGIF1-/-; TGIF2 +/r	13	**6.02%**	6.25%
Cre+; TGIF1+/-; TGIF2 r/r	11	**5.09%**	6.25%
Cre+; TGIF1+/-; TGIF2 +/r	36	**16.67%**	12.50%
Cre+; TGIF1-/-	2	**0.93%**	3.13%
Cre+; TGIF1+/-	17	**7.87%**	6.25%
Cre+; TGIF2 r/r	8	**3.70%**	3.13%
Cre+; TGIF2 +/r	20	**9.26%**	6.25%
Cre+	8	**3.70%**	3.13%
Cre- embryos	101	**46.76%**	50%
Total	216		

Supplementary Figure 1. *Tgif2* is expressed in embryonic and adult pancreatic tissues. (**a**) IF analysis of TGIF2 in whole-mount E8.5 mouse embryo. Overlay of TGIF2 (red) and FOXA2 (green) stainings in ventral (arrow) and dorsal (black asterisk) foregut endoderm. Scale bars, 50 µm. (**b**) *In situ* hybridisation analysis for *Tgif2* on cryosections of mouse pancreas at E18.5. Endocrine islets are demarcated by yellow dotted lines. end, endocrine. Scale bars, 100 µm. (**c**) Co-staining of TGIF2 and INSULIN in mouse adult pancreas. On the left, merged fluorescence and Hoechst; on the right, micrograph without the Hoechst channel (blue). TGIF2 expression is maintained in the adult pancreas, being

abundant in the endocrine islets and expressed at lower level in the surrounding acinar cells (arrows). ***(*d*)*** Table summarizing the observed and expected frequency of genotypes (%) of 216 embryos obtained from breeding of *Tgif1*$^{+/-}$*;Tgif2*$^{lox/+}$*;Sox2-Cre*$^{+}$ X *Tgif1*$^{+/-}$*;Tgif2*$^{lox/+}$ mice. Embryos were dissected between embryonic stages E7.5 – E12.5. No double null embryos were found in our background. Given that Sox2-Cre is active in all epiblast cells[1], it cannot be ruled out that *Tgif2* effects in the endoderm might be the results of both cell-autonomous and non-cell-autonomous mechanisms. r, recombined.

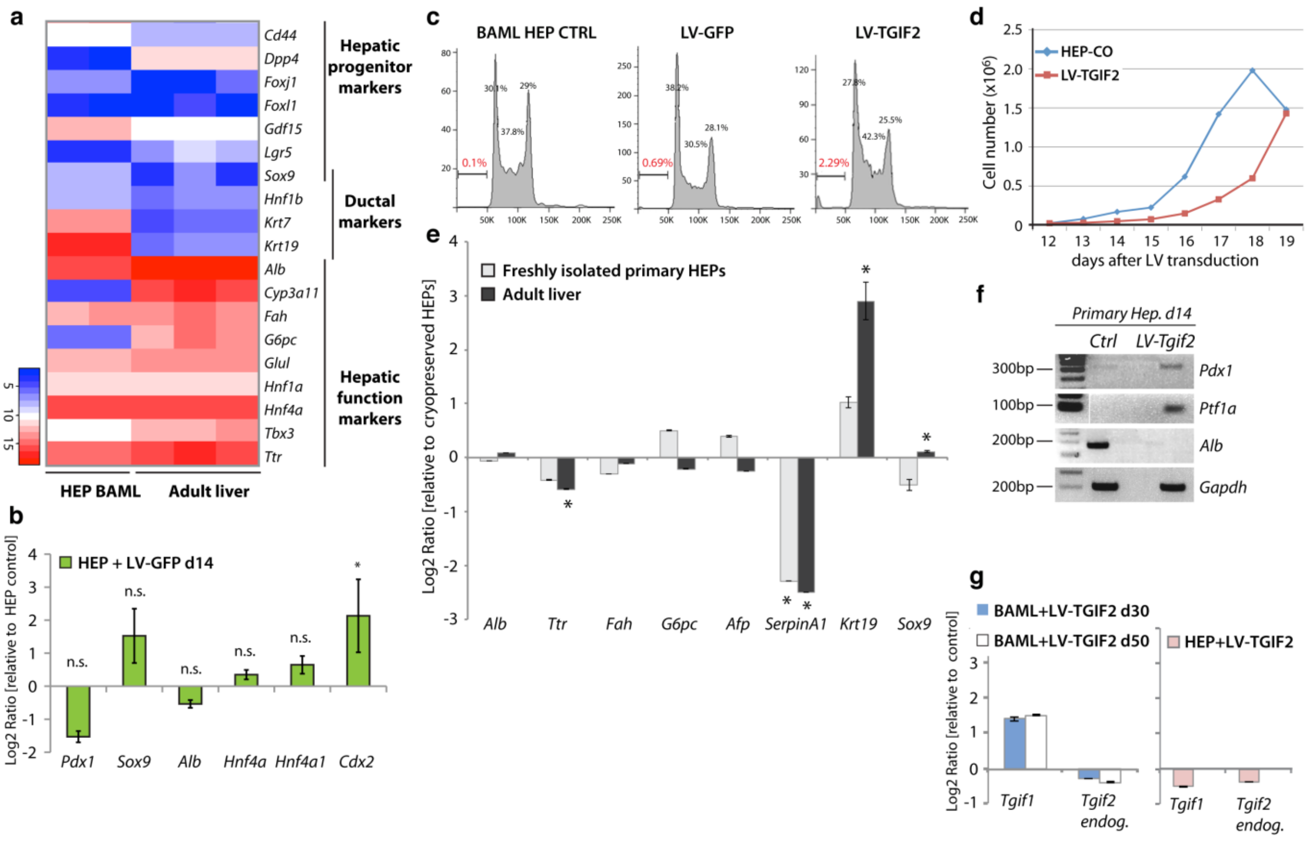

Supplementary Figure 2. Characterization of LV-transduced HEP cells.

(**a**) Heat map of expression levels of selected hepatic progenitor, ductal and hepatic function markers in BAML HEP control cells and adult liver tissue. BAML HEP displayed several mature hepatic molecular features similar to adult mouse liver and were not enriched for hepatic progenitor markers. Colors represent high (red) or low (blue) expression levels. (**b**) RT-qPCR analysis of BAML HEP cells transduced with LV-GFP and cultured for 14 days. Neither induction of pancreatic transcripts nor repression of hepatic transcripts was detected upon ectopic expression of the control LV-GFP lentivirus. Data were normalized to that of *Sdha* and shown as 2-log fold changes compared to non-transduced HEP cells. Values shown are mean ± SEM. ns, not significant. (**c**) Flow cytometric analysis of cell cycle using propidium iodide (PI) DNA

staining of BAML HEP cells at day 4 after transduction with LV-TGIF2 or LV-GFP and non-transduced HEP control (CTRL). The x-axis shows fluorescence intensity (PI) and y-axis represents cell count. % of cells in each cell cycle phase is indicated. At day 4 post-transduction, LV-TGIF2 cells exhibited a lowered fraction of G1 cells and an increased percentage of cells in S-phase with a minor increase of cells in apoptosis (in Red). This is in line with the fact that cell division is not required in many cases of somatic lineage reprogramming. (**d**) Growth curve of BAML HEP + LV-TGIF2 and control (HEP-CO) cells. Cells were seeded 12 days after transduction at $2.5x10^4$ cells/well density and counted daily until confluence was reached. **(e)** RT-qPCR analysis of representative hepatocyte- and biliary epithelial cell-specific markers in cryopreserved mouse hepatocytes (HEP), freshly isolated hepatocytes and adult liver. Overall, cryopreserved HEPs reflected a true well-differentiated hepatic phenotype, displaying same morphology and level of expression of crucial hepatic genes comparable to freshly isolated hepatocytes. The biliary markers, Cytokeratin (Krt) 19 and Sox9, were expressed in adult liver but not in hepatocytes as expected. Data were normalized to *Sdha* and represented as Log2-expression ratio relative to cryopreserved HEP cells. Values shown are mean ± sem. *P< 0.05. **(f)** Semiquantitative RT-PCR analysis of indicated pancreatic and hepatic marker genes in primary hepatocytes transduced with LV-TGIF2 at d14 after transduction and control (Ctrl) cells cultured for the same period of time and in the same culture conditions. DNA size marker (100bp ladder) is shown on the left. **(g)** RT-qPCR analysis of endogenous *Tgif2* and *Tgif1* gene expression in BAML and primary HEP cells transduced with LV-TGIF2 at the indicated time points after transduction. No significant changes in Tgif2 and Tgif1 expression levels were measured. Data were normalized to

Sdha and represented as Log2-expression ratio between LV-TGIF2 transduced cells and control cells. Values shown are mean ± s.e.m.

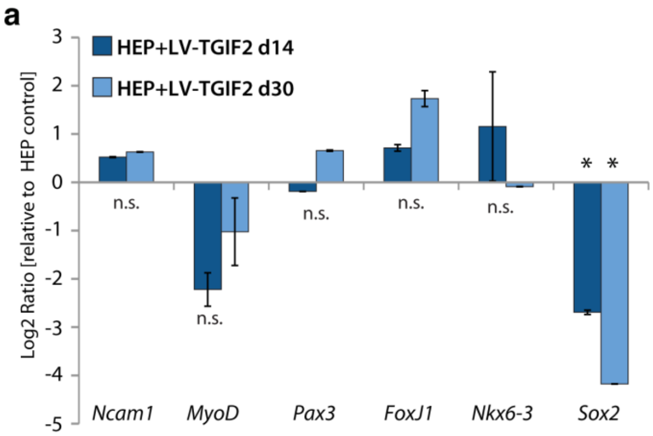

b

Lineage	Gene Symbol	Log-FC	p value
Neuronal progenitors	Foxg1	0.62	0.67
	Gpm6a	-1.30	0.06
	Lhx2	1.34	0.32
	Ncam1	0.53	0.41
	Rfx4	-0.33	0.80
	Sox1	-0.68	0.48
	Tox3	1.50	0.23
Cardiac / Muscle progenitors	Hand1	-0.40	0.72
	Hand2	-0.12	0.95
	Mef2c	0.68	0.51
	Myod1	-0.59	0.37
	Nkx2-5	-0.64	0.76
	Pax3	-0.34	0.20
	Pax7	-0.14	0.96
	Tbx5	-0.57	0.65
	Tbx20	0.09	0.99
Thyroid	Foxe1	-0.52	0.84
	Pax8	0.02	1.00
	Tshr	-0.10	0.98
	Tg	-1.59	0.04
Lung	Fgfr2	-0.47	0.20
	Foxj1	0.84	0.69
	Foxp2	-0.02	1.00
	Sftpc	-0.01	1.00

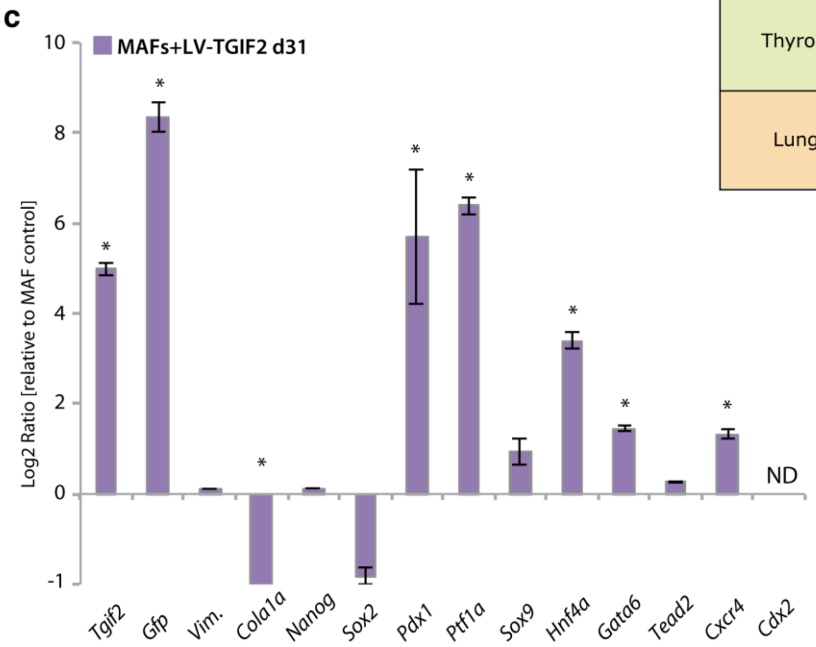

Supplementary Figure 3. Tgif2 activity in non-endodermal lineage cells.

(a) RT-qPCR analysis of representative lineage-specific markers in BAML HEP cells transduced with LV-TGIF2 at the indicated time points after transduction. Data were normalized to *Sdha* and represented as Log2-expression ratio between BAML HEP + LV-TGIF2 cells at d14 or d30 and control cells. Values shown are mean ± s.e.m. *$P<$ 0.05. ns, not significant. (**b**) Table illustrating differential expression values of key lineage-specific markers in BAML HEP + LV-TGIF2 d14 cells *versus* BAML HEP

control cells. Data are from the microarray analysis (see Fig. 5) and shown as Log2 Fold-Change (Log-FC). The p-value was corrected for multiple comparisons using the Benjamini-Hochberg method. **(c)** Ectopic *Tgif2* expression in mouse adult skin fibroblasts (MAF) induces *Pdx1* and *Ptf1a* expression and to a lesser extent endodermal genes, such as *Hnf4a*, *Gata6* and *Cxcr4*. RT-qPCR characterization of MAFs transduced with LV-TGIF2 (see Fig. 3) at d31 after transduction. Expression of both *Tgif2* and *Gfp* transgenes was measured along with indicated fibroblast and endodermal marker genes. Data were normalized to *Sdha* and represented as Log2-expression ratio between MAFs + LV-TGIF2 cells at d31 and control cells cultured for the same period of time and in the same culture conditions. Values shown are mean ± s.e.m. *$P < 0.05$. ND, not determined.

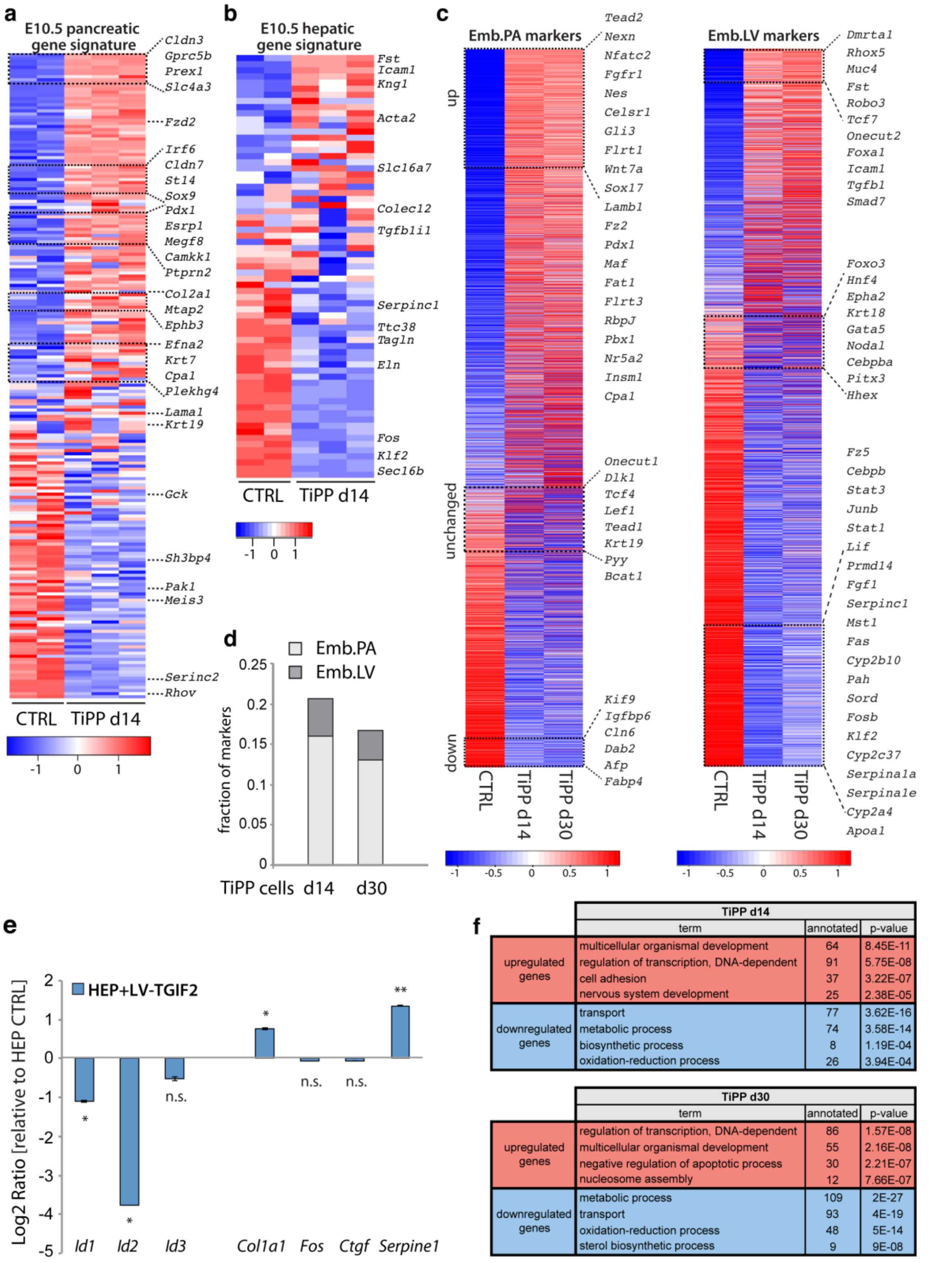

Supplementary Figure 4. *Tgif2*-expressing reprogrammed cells are closer to embryonic pancreas than to embryonic liver. **(a)** Heat map of E10.5 pancreatic

progenitor signature genes in TiPP cells and HEP control (CTRL) population. A total of 153 genes were defined as pancreatic progenitor-specific signature genes for their high expression values (FPKM >10) in mouse E10.5 pancreatic progenitors and low expression values (FPKM <4) in E10.5 hepatic progenitors in RNA-Seq datasets[2]. This E10.5 pancreatic gene signature was then compared to TiPP d14 and control population transcriptome. (**b**) Heat map of E10.5 hepatic progenitor signature genes in TiPP cells and HEP control (CTRL) population. A total of 69 genes were defined as hepatic progenitor-specific signature genes for their high expression values (FPKM >10) in mouse E10.5 hepatic progenitors and low expression values (FPKM <4) in E10.5 pancreatic progenitors in RNA-Seq datasets[2]. This E10.5 hepatic gene signature was then compared to TiPP d14 and control population transcriptome. (**c**) TiPP gene expression of mouse embryonic pancreas (Emb.PA) and embryonic liver (Emb.LV) markers. Emb.LV microarray data were taken from Gene Expression Omnibus (http://www.ncbi.nlm.nih.gov/geo), accession number GSE67406 ("E13_0day samples": GSM1646389, GSM1646392). The data were batch-normalized with our microarray data generated in the present study. Marker genes for Emb.PA were defined to be up-regulated genes in Emb.PA compared to Emb.LV (p-value < 0.05 and FC > 2; 4763 genes). Accordingly, marker genes for Emb.LV were defined to be up-regulated genes in Emb.LV compared to Emb.PA (p-value < 0.05 and FC > 2; 3990 genes). To evaluate the expression of Emb.PA and Emb.LV marker genes in the TiPP cells, differentially regulated genes (p-value < 0.05 and FC > 2) were calculated for TiPPd14 and TiPPd30 compared to HEP control cells. The heatmaps show the median expression levels in HEP controls and TiPPd14 and TiPPd30 cells. The rows are ordered according to the mean

fold-change of TiPP cells relative to HEP control, and columns are ordered by hierarchical clustering of the expression values. Gene expression across rows in the heatmap are colored according the z-score, such that the mean expression of each row is set to white color and expression values higher or lower than the mean are graded towards red or blue, respectively. (**d**) Proportion of Emb.PA or Emb.LV markers that are up-regulated in TiPP cells. Marker genes for Emb.PA and Emb.LV were defined as described above. To demonstrate the expression of Emb.PA and Emb.LV marker genes in the TiPP cells, differentially regulated genes (p-value < 0.05 and FC > 2) were calculated for TiPPd14 and TiPPd30 compared to HEP control cells. The stacked barplot shows the fractions of up-regulated genes in TiPP cells (relative to HEP CTRL) found in the EmbPA and EmbLV markers, respectively. The TiPP cells demonstrate a higher fraction of expression in the EmbPA marker list. **(e)** RT-qPCR analysis of selected TGF-β and BMP signaling target genes. Data were normalized to *Sdha* and represented as Log2-expression ratio between HEP + LV-TGIF2 and CTRL cells. Values shown are mean ± s.e.m. (n=3) **P*< 0.05. (**f**) GO "biological process" enrichment analysis of differentially regulated genes in HEP control and TiPP cells (*P*< 0.05 and FC >2 or <-2). The four non-redundant GO categories with highest p-values are shown per each group. For complete GO annotation lists see Supplementary Data 1.

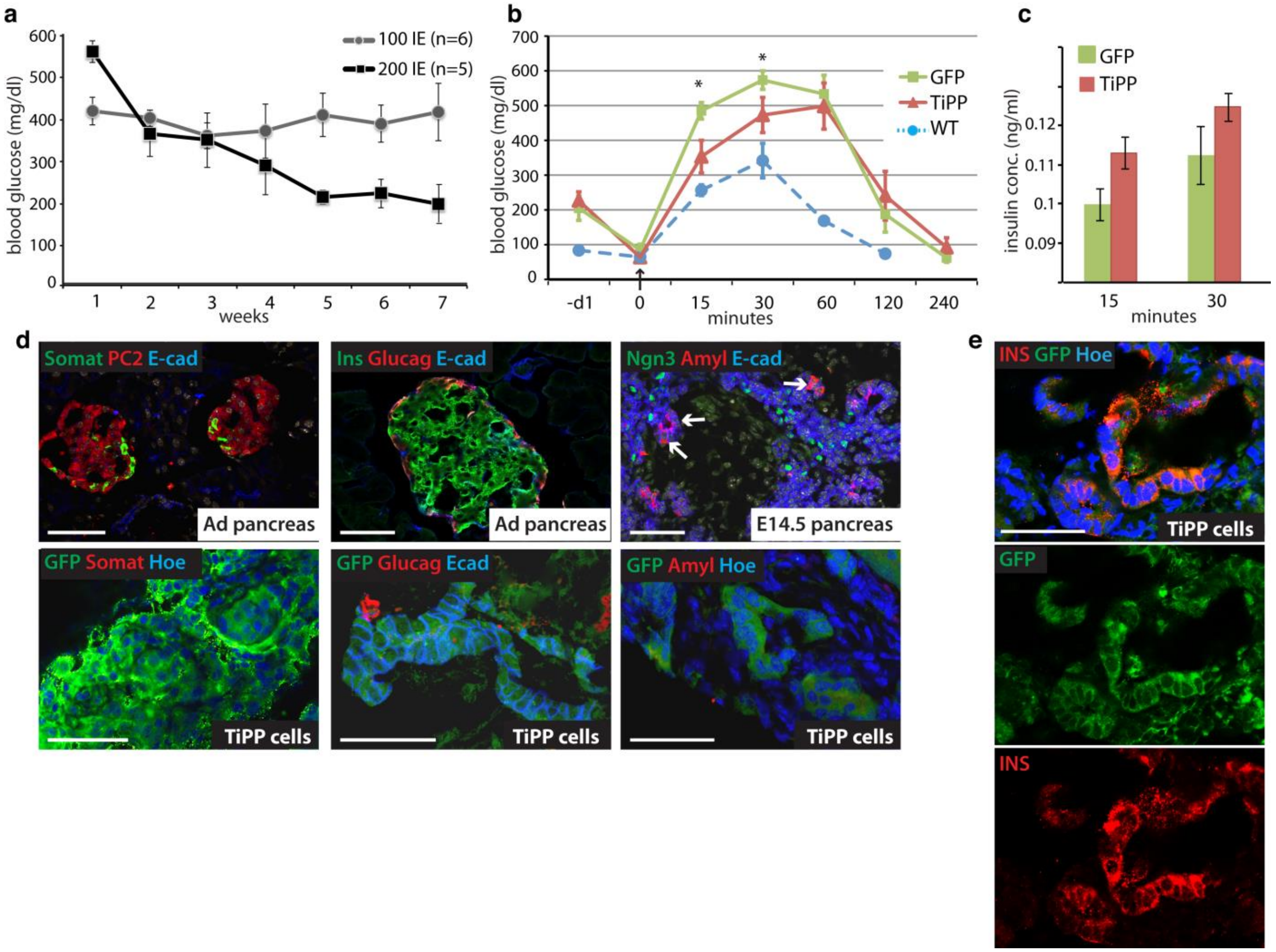

Supplementary Figure 5. Characterization of TiPP cells differentiation potential *in vivo*. (**a**) Transplantation of 100 and 200 mouse islet equivalents (IE) under the KC of hyperglycemic Akita mice. Blood glucose levels were measured under non-fasting conditions before transplantation, twice a week after transplantation. Normoglycemia is defined as blood glucose level below 200 mg/dl in wild-type littermate mice under nonfasting conditions (see also Fig. 6). Values shown are mean ± SD. (**b**) Glucose challenge assay in Akita mice engrafted with TiPP cells (red line; n=4) or LV-GFP BAML HEP (green line; n=3) cells, as control. Intraperitoneal glucose tolerance test (GTT) was performed two weeks after transplantation in heterozygotes Akita females,

which display moderate hyperglycemia (blood glucose level above 200 mg/dl after starvation) but no overt diabetes. Blood glucose was measured before (–d1) and after overnight starvation (d0, baseline level), and then at the indicated timepoints after glucose injection. Blood glucose levels in C57/BL6 wild-type female mice during GTT test were used as reference (blue dotted line). Despite the limited number ($1x10^6$) of engrafted TiPP cells, our results showed that TiPP cells improved blood glucose profiles in non-fasting conditions of Akita mice (see Fig. 6a) and are also glucose-responsive, as indicated by increased glucose clearance and insulin release (Fig. S5c). Data are expressed as mean ± S.D. Statistics by two-tailed t-test. (**c**) Serum insulin concentration was measured during the glucose tolerance test (see Fig. S5b) at the indicated time points after overnight starvation. Results are presented as mean ± S.D. (**d**) Top panel, IF analysis of adult and E14.5 mouse pancreas for the indicated pancreatic endocrine and exocrine markers. Adult mouse islets were stained for somatostatin (Somat), Prohormone Convertase 2 (PC2), insulin (Ins) and glucagon (Glucag). In E14.5 mouse pancreas, arrows indicate Amylase (Amyl) staining in exocrine progenitor cells. Bottom panel, antibodies against the same endocrine and exocrine markers were tested on cryosections of mouse kidneys transplanted with TiPP cells. Scale bars, 50 µm. (**e**) IF stainings for Insulin and GFP, shown in Figure 6b, presented as single channel images. Scale bars, 50 µm.

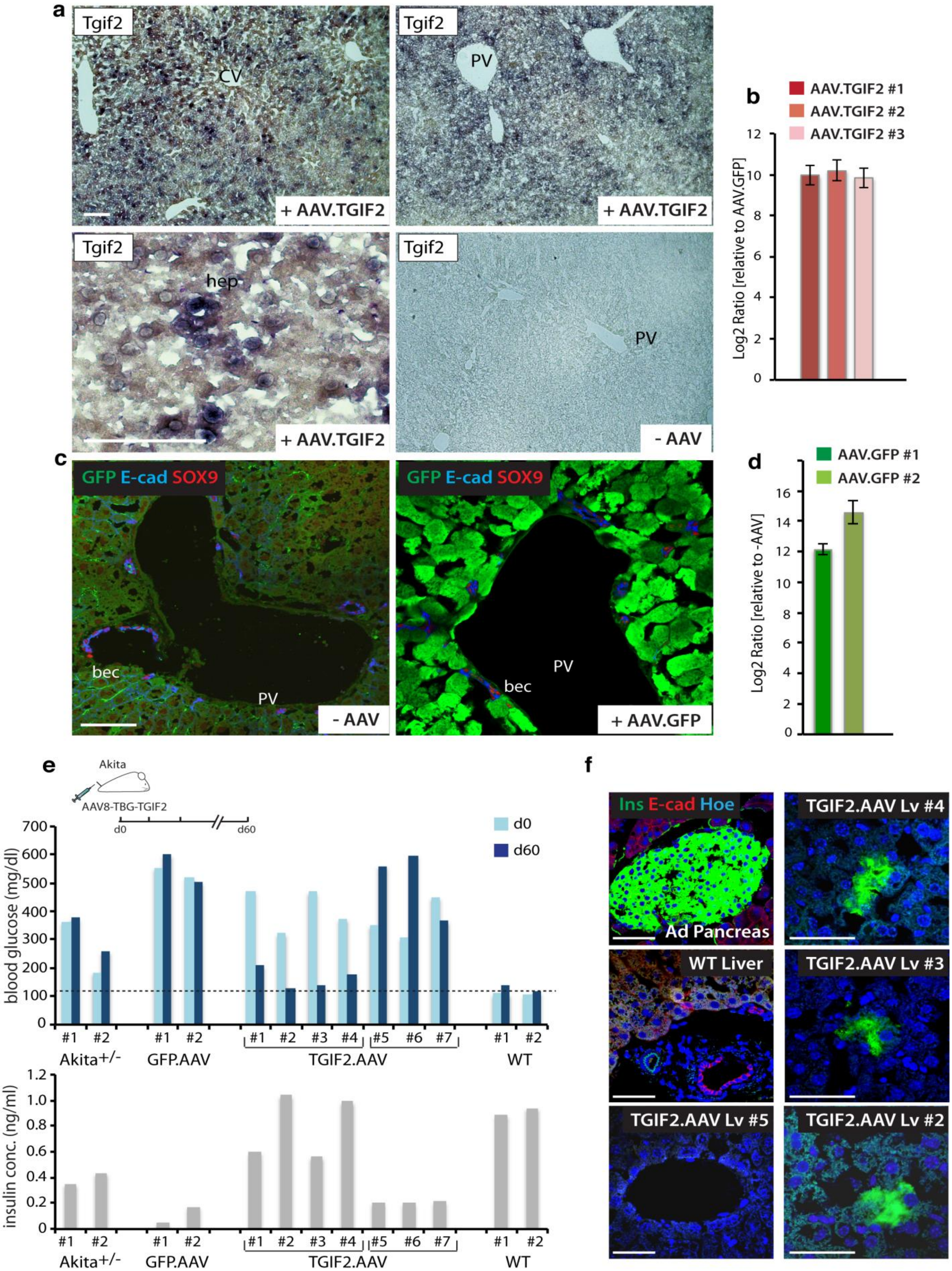
a
Tgif2
CV
+ AAV.TGIF2
Tgif2
PV
+ AAV.TGIF2
Tgif2
hep
+ AAV.TGIF2
Tgif2
PV
- AAV
b
AAV.TGIF2 #1
AAV.TGIF2 #2
AAV.TGIF2 #3
Log2 Ratio [relative to AAV.GFP]
c
GFP E-cad SOX9
bec
PV
- AAV
GFP E-cad SOX9
bec
PV
+ AAV.GFP
d
AAV.GFP #1
AAV.GFP #2
Log2 Ratio [relative to -AAV]
e
Akita
AAV8-TBG-TGIF2
d0
d60
blood glucose (mg/dl)
Akita+/-
GFP.AAV
TGIF2.AAV
WT
insulin conc. (ng/ml)
f
Ins E-cad Hoe
Ad Pancreas
TGIF2.AAV Lv #4
WT Liver
TGIF2.AAV Lv #3
TGIF2.AAV Lv #5
TGIF2.AAV Lv #2

Supplementary Figure 6. Characterization of AAV-mediated Tgif2 *in vivo* liver expression. (**a**) Characterization of AAV-mediated Tgif2 *in vivo* liver expression. *In situ* hybridisation with *Tgif2* antisense RNA probe on liver cryosections of control (-AAV) and AAV.TGIF2-injected adult mice at d30. Robust induction of *Tgif2* transcript was observed only in the liver of AAV.TGIF2-injected animals throughout the parenchyma. *Tgif2* transgene expression was still detectable at d60 after AAV injection (data not shown). Scale bars, 100 µm. CV, central vein; hep, hepatocytes; PV, portal vein. (**b**) Representative RT-qPCR of *Tgif2* in AAV.TGIF2- and AAV.GFP-injected adult livers at d30. Data were normalized to 36B4 reference gene and represented as Log2-expression ratio between AAV.TGIF2-inj. and AAV.GFP-inj. adult livers. Values shown are mean ± sem. n=3. (**c**) IF staining of AAV-injected (+AAV.GFP) and uninjected (-AAV) adult mouse livers for GFP, SOX9 and E-cad. SOX9 marks biliary epithelial cells (bec) in both AAV-GFP-injected and uninjected adult livers, but it is not expressed in hepatocytes. This indicates that AAV injection itself (*e.g.* AAV.GFP) did not induce activation of SOX9 in hepatocytes. PV, portal vein. Scale bars, 100 µm. (**d**) Representative RT-qPCR of *GFP* in AAV.GFP-injected at d30 and uninjected adult livers. Data were normalized to 36B4 and represented as Log2-expression ratio between AAV.GFP-inj. and uninjected (-AAV) adult livers. Values shown are mean ± s.e.m. n=2. **(e)** Schematic of the AAV-mediated experimental strategy to express *Tgif2 in vivo* in adult mouse liver of mild hyperglycemic Akita heterozygotes diabetic mice. AAV-injected and control animals were examined at the indicated time points after *i.v.* injection. Top panel, blood glucose levels of control and treated animals measured under non-fasting conditions before AAV-injection (time point: d0) and at the end of the experiment (d60). Blood glucose levels

were markedly reduced, being almost restored to normoglycemia in 4 out of 7 Akita$^{+/-}$ mice (#1 to #4) after AAV.TGIF2-injection. By contrast, blood glucose levels remained elevated in non-treated diabetic Akita$^{+/-}$ and AAV.GFP-injected Akita$^{+/-}$ mice. WT uninjected mice mantained stable normoglycemia (as marked by black dotted line). Bottom panel, serum insulin levels measured in the same control and treated animals under non-fasting conditions at time point d60 after AAV-injection. Importantly, the group of AAV.TGIF2-injected Akita mice (#1 to #4), which displayed reduced blood glucose levels at d60, also exhibited higher levels of circulating insulin when compared to untreated diabetic Akita or AAV.GFP-injected mice. Individual values per animal are shown. (**f**) IF staining of AAV.TGIF2 injected-adult liver from Akita$^{+/-}$ mice for insulin (INS) and E-cadherin (E-cad). Adult mouse pancreas and adult WT liver were included as positive and negative controls, respectively, in the IF staining. Insulin-positive cells were found exclusively in the liver of AAV.TGIF2-injected Akita mice (#1 to #4), which displayed reduced blood glucose levels and higher levels of circulating insulin at d60. Scale bars, 50 μm. Together, these results are consistent with the kidney capsule transplantation of TiPP reprogrammed cells and suggest that environmental cues, such as a hyperglycemic state, promote maturation of the Tgif2-induced pancreatic progenitor-like cells.

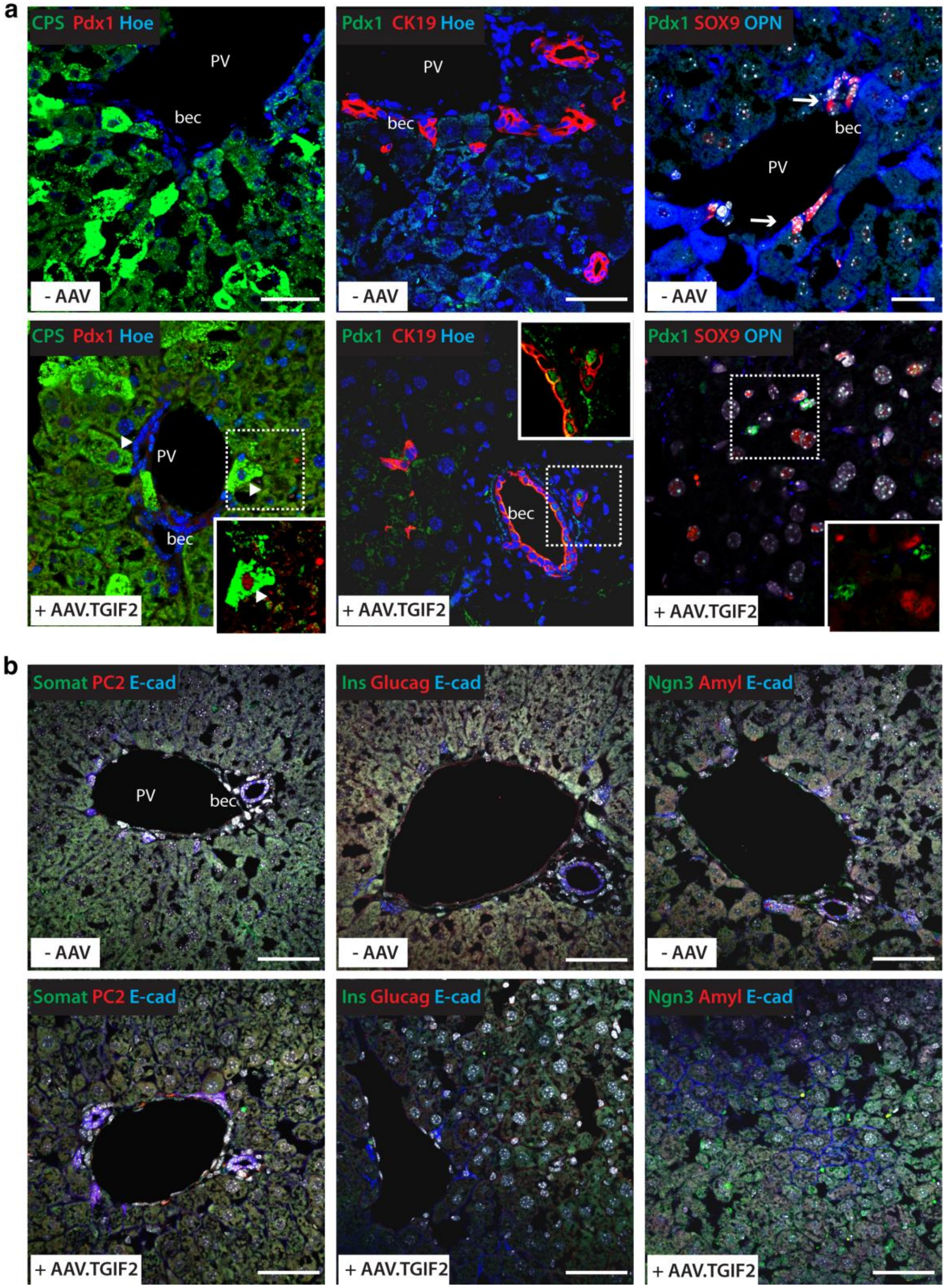

Supplementary Figure 7. Characterization of AAV8.TBG-Tgif2-injected livers. (**a**) IF staining of AAV-injected (+AAV.TGIF2) and uninjected (-AAV) adult mouse livers

for the periportal hepatocyte marker, Carbamoyl Phosphate Synthase 1 (CPS1), biliary epithelial markers, cytokeratin 19 (CK19) and osteopontin (OPN), and pancreatic markers (PDX1 and SOX9). PDX1-positive cells are detected in the periportal zone of AAV-TGIF2-injected adult livers and co-stain with CPS1 (see arrowheads). CK19 marks biliary epithelial cells (bec); in inset, CK19- and PDX1-double positive cells in AAV-TGIF2-injected adult livers. In AAV-TGIF2-injected adult livers activation of SOX9 and PDX1 expression in hepatocytes (see inset) is not accompanied by OPN, ruling out the acquisition of bec fate. Arrows indicate expression of OPN in biliary epithelial cells (bec) together with SOX9. Biliary epithelial cells (bec); PV, portal vein. Scale bars, 50 µm. (**b**) IF staining of AAV-injected (+AAV.TGIF2) and uninjected (-AAV) adult mouse livers for somatostatin (Somat), Prohormone Convertase 2 (PC2), insulin (Ins) glucagon (Glucag), Ngn3, Amylase (Amyl) and Ecad. AAV-TGIF2-injection in WT adult livers did not induce pancreatic differentiation markers. Scale bars, 50 µm.

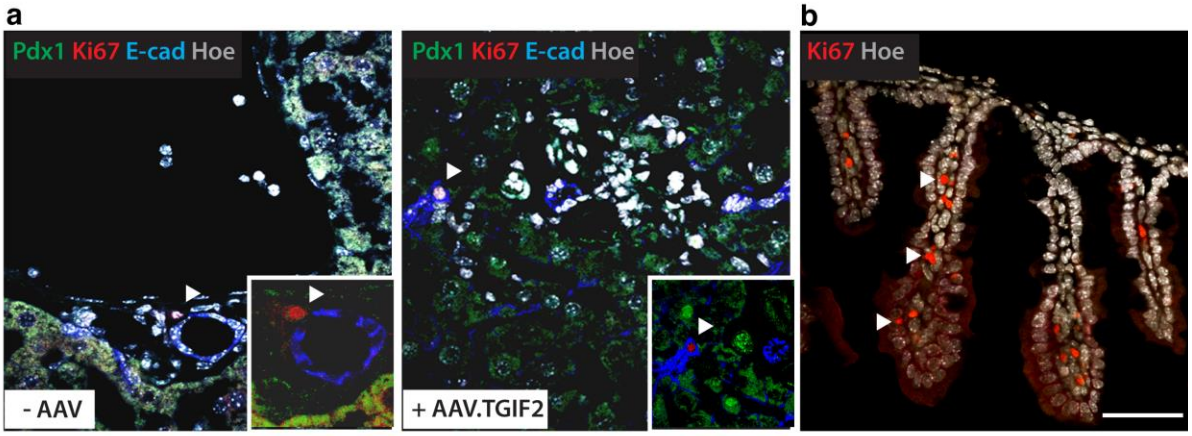

Supplementary Figure 8. Characterization of proliferation in AAV8.TBG-Tgif2-injected livers. (**a**) IF staining of AAV-injected (+AAV.TGIF2) and uninjected (-AAV) adult mouse livers for Ki67, PDX1 and E-cadherin (E-CAD). Very rare Ki67-nuclei were found in both uninjected and AAV-injected livers (see arrowheads), ruling out the possibility that hepatocytes enter the cell cycle upon AAV stimuli. (**b**) IF staining of adult mouse intestinal epithelium was used as positive control for Ki67 staining. Arrowheads indicate cells positive for the proliferation marker Ki67 in red. Scale bars, 50 µm.

Supplementary Table 1. Primer sequences used for quantitative real-time PCR

Gene symbol	Forward primer	Reverse primer
Afp	CGAGGAGTGTTGCCAAGGAAA	CAGAAGCCTAGTTGGATCATG
Alb	TGCTGCTGATTTTGTTGAGG	GCAGCACTTTTCCAGAGTGG
Cdx2	AAACCTGTGCGAGTGGATG	CTGCGGTTCTGAAACCAAAT
Celsr1	TGGTACCAGGGCAGGCTGTGCTTC	CCAGTGAAGCGCTGAGGGTGTG
Cebpa	CAAGGCCATGGGCAACTGCG	TCTGGGTCTCCAGCCAGGGG
Celsr3	CCAGGCCAAGTCACACTTTTG	TAGGGATGGGCCATTGTGAGT
Cldn3	CGTCAGTTTTCGAAGGGCAG	ATGGCTGCTGGACTTGAACC
Cola1a	CTGACGCATGGCCAAGAAGA	CTCGGGTTTCCACGTCTCAC
Cxcr4	CTCTGAGGCGTTTGGTGCTCCG	TCCCGGAAGCAGGGTTCCTTGT
Dlc1	CGCCTTGACAGGATCCGGCG	TGTCCCGGTTAGCGGCTCCA
Fah	GGCCCTGGGAACAGATTCGGA	GTCTCGTGCGCTCCAGTCGT
Fgfr1	TGCCTGAACAAGATGCACTCCCA	TCTGGGGATGTCCAGTAGGGAGC
Foxa2	CATCCGACTGGAGCAGCTA	GCGCCCACATAGGATGAC
FoxJ1	CGAGCTGGGGACAGAGAACCG	GAACACGAATGTGAGGCCTGGCT
Gata6	GGTCTCTACAGCAAGATGAATGG	TGGCACAGGACAGTCCAAG
Hex	GAGGTTCTCCAACGACCAGA	GTCCAACGCATCCTTTTTGT
Hnf4a	AACCACGCTACTTGCCTTTGCT	TCTGATGGGACACAGCCTACTTCT
Hnf4a1	GAAAATGTGCAGGTGTTGACCA	AGCTCGAGGCTCCGTAGTGTTT
Insm1	GCCCAGGTGTTCCCCTGCAA	AGGCCCGGGGAGCTGTAGAA
Isl1	GCGGCCTCTGCAAATGGCAG	CTCCGGCTGCTTGTGGACGT
Mafa	GAGGTGAAGAAGGAGCCGCCC	CTGAGGGGCGTCGAGGACAG
Lgr5	CAGTGTTGTGCATTTGGGGG	CAAGGTCCCGCTCATCTTGA
Myod1	GATGGCATGATGGATTACAGCGGC	CGCGCCGCCTCACTGTAGTA
Ncam1	CAGAGCGCTCGCCTCTGAGT	ACGTGGTCACTGCCGGATGG
Nexn	AGGAGGCGAGAAGGCACATGGTAA	CTGAGTTTGAGTTTTCCTGGGCGGT
Nestin	GCTGTGGAAGCCCTGGAGCA	TCAGCCTCCAGCAGAGTCCTGT
Neurod1	AAGGCAAGGTGTCCCGAGGC	CATCAGCCCGCTCTCGCTGT
Nkx6.3	GCCATGCAGCAACACTCCAGAC	TGGTGGCCCGTGAAGGTAGG
Pax6	CAACCTGCCTATGCAACCCCCA	GGGCAGCATGCACGAGTACGA
Pdx1	CCACCAAAGCTCACGCGTGGA	GGCGGGGCCGGGAGATGTATT
Prox1	CCGACATCTCACCTTATTGAG	TGCGAGGTAATGCATCTGTTG
Ptf1a	TTCCTGAAGCACCTTTGACAGA	ACGGAGTTTCCTGGACAGAGT
Ror2	CTATATGTGCGGCTCGGTCC	AGGTGCCGATCATGGTGAAG
Scrib	AGTCTGCAGAGACCACGGGTCG	ATGGCTGGATCACCCCCGGTT
Sdha	TGTTCAGTTCCACCCCACA	TCTCCACGACACCCTTCTGT
Serpina1	GAAGCTGCAGCAGCTACAGTC	TGTGGGATCTACCACTTTTCC
Sox17	GGTCTGAAGTGCGGTTGG	TGTCTTCCCTGTCTTGGTTGA
Sox9	AGACTCACATCTCTCCTAATGCT	ACGTCGGTTTTGGGAGTGG
Sox2	CTCTGCACATGAAGGAGCAC	CTCCGGGAAGCGTGTACTTA
Tead2	CTGGACAGGTAGCGAGGAAG	GTTCCGGCCATACATCTTGC
Tgif1	CGCGCCCGCGTGTCAAT	TTCTGATTCAAGGGGTGGGGCAC

Tgif2	CTATCTGCACCGCTACAACG	GGGCATTGATGAACCAGTTAC
Tgif2 3'UTR	GGCGAGAATGGCTATTTGTAA	AGCCTGCTGGGTCTCCTAC
Ttr	CGTTCCATGAATTCGCGGATGTGGT	GCAGGGCTGCGATGGTGTAGT
Vangl2	CAGCTCCCGGAAGCACAGGGAC	GTGCCCGTGACCACCGTTGT
Vimentin	GGATCAGCTCACCAACGACA	AAGGTCAAGACGTGCCAGAG

Supplementary Table 2. List of primary antibodies and dilution used.

Antibody	Catalog number	Host Species	Dilution
anti-ALBUMIN	DakoCytomation, A0001	rabbit	1:100
anti-E CADHERIN	Invitrogen, 13-1900	rat	1:1000
anti-C PEPTIDE 1	BCB Consortium, AB1044	rabbit	1:1500
anti-CPS1	Abcam, ab3682	rabbit	1:500
anti-CK19	Abcam, ab133496	rabbit	1:200
anti-EPCAM	Sigma	rabbit	1:150
anti-FOXA2	Abcam, Ab408749	rabbit	1:200
anti-GFP	Aves, GFP-1020	chicken	1:500
anti-GFP	Abcam, ab290	rabbit	1:500
anti-GLUTAMINE SYNTHETASE	BD Transduction labs, 410517	mouse	1:500
anti-GLUT2	Santa Cruz, sc-7580	goat	1:200
anti-HNF4a	Santa Cruz, sc-6556	goat	1:100
anti-INSULIN	Invitrogen, 18-0067	guinea pig	1:250
anti-Ki67	ThermoFisher, RM9106	rabbit	1:200
anti-NKX6.1	Hybridoma Bank, F55A10	mouse	1:150
anti-OSTEOPONTIN	Abcam, ab 166709	mouse	1:400
anti-PDX1	Abcam, ab 408749	rabbit	1:200
anti-PDX1	Abcam, ab 47308	guinea pig	1:500
anti-Prohormone Convertase PC2	Millipore, AB15610	rabbit	1:100
anti-PROX1	Chemicon, AB5475	rabbit	1:500
anti-PROX1	RELIATech GmbH, 102PA32S	rabbit	1:200
anti-SOMATOSTATIN	Santa Cruz, sc-7819	goat	1:100
anti-SOX17	R&D Systems	goat	1:100
anti-SOX9	M. Wegner, Nürnberg University	rabbit	1:500
anti-SOX9	Millipore, AB5535	rabbit	1:500
anti-TGIF2	Abnova, H00060436-A01	mouse	1:200
anti-TGIF2	Abnova, H00060436-M01, clone 4C10	mouse	1:200
anti-UCN3	P.E. Sawchenko, Salk Institute	rabbit	1:300

Supplementary References

1 Hayashi, S., Lewis, P., Pevny, L. & McMahon, A. Efficient gene modulation in mouse epiblast using a Sox2Cre transgenic mouse strain. *Mech Dev* **119**, 1:S97-101 (2002).

2 Rodríguez-Seguel, E. *et al.* Mutually exclusive signaling signatures define the hepatic and pancreatic progenitor cell lineages divergence. *Genes Dev.* **27**, 1932-1946 (2013).
